# Targeted Cooperative Actions Shape Social Networks

**DOI:** 10.1371/journal.pone.0147850

**Published:** 2016-01-29

**Authors:** Lucas Wardil, Christoph Hauert

**Affiliations:** Department of Mathematics, University of British Columbia, Vancouver, BC, Canada; University of Waterloo, CANADA

## Abstract

Individual acts of cooperation give rise to dynamic social networks. Traditionally, models for cooperation in structured populations are based on a separation of individual strategies and of population structure. Individuals adopt a strategy—typically cooperation or defection, which determines their behaviour toward their neighbours as defined by an interaction network. Here, we report a behavioural experiment that amalgamates strategies and structure to empirically investigate the dynamics of social networks. The action of paying a cost *c* to provide a benefit *b* is represented as a directed link point from the donor to the recipient. Participants can add and/or remove links to up to two recipients in each round. First, we show that dense networks emerge, where individuals are characterized by fairness: they receive to the same extent they provide. More specifically, we investigate how participants use information about the generosity and payoff of others to update their links. It turns out that aversion to payoff inequity was the most consistent update rule: adding links to individuals that are worse off and removing links to individuals that are better off. We then investigate the effect of direct reciprocation, showing that the possibility of direct reciprocation does not increase cooperation as compared to the treatment where participants are totally unaware of who is providing benefits to them.

## Introduction

Networks are powerful abstractions of human interactions [1]. Populations can be represented as graphs where individuals occupy nodes and links indicate their interaction partners. Cooperative interactions are argued to be essential to construct new levels of organization, integrating individuals into higher level entities [2]. However, because cooperators incur cost *c* to provide benefit *b* to others, the temptation of receiving benefits without paying the costs endangers the sustainability of cooperative interactions. This represents the donation game, a particular instance of a social dilemma known as Prisoner’s Dilemma [3]. Depending on the cost to benefit ratio [4], cooperation can thrive if there exists a mechanism, which yields positive assortment among cooperators. One such mechanism is network reciprocity [5]. In this setting, individuals typically adopt the same strategy in all interactions with their neighbours as determined by the network. Theoretical studies have shown that static networks promote cooperation in humans through the formation of clusters of cooperators [6, 7]. However, static networks seem unnatural in humans. Different from other animals, humans cooperate with strangers in much more volatile social networks [8]. Hence, human cooperation is more naturally described by dynamical network models, where individuals may choose with whom they want to interact and what behaviour—cooperation or defection—they want to adopt in each interaction. Theoretical studies have shown that dynamical networks promote cooperation under various circumstances through positive assortment between cooperators [9–11].

In recent years, behavioural experiments have been designed to test theoretical predictions of the effect of networks on human cooperation. Surprisingly, there has been no consistent experimental evidence that static networks are capable of promoting human cooperation [12–15]. At the same time, while theoretical investigations have been tacitly assuming imitation rules based on payoff differences [16], experiments have shown that imitation rules based on payoff comparisons are not as universal as expected [17], suggesting more idiosyncratic update strategies. In contrast to static networks, behavioural experiments confirm that dynamic networks, which first and foremost admit partner choice, promotes human cooperation [18–20]. In one experimental study, dynamic partner updating significantly increased the level of cooperation, the average payoffs to players, and the assortativity between cooperators [19]. The results are robust over a wide range of parameters controlling the ratio between link updates and strategy updates. Links were allowed to be broken unilaterally, while new links required mutual consent to be established. A related study showed that cooperation was promoted most at intermediate levels of rewiring rates [20]. However, in this experiment links could be added without requiring mutual consent.

Partner choice can be viewed as a biological market governed by the supply and demand of desirable partners and implemented as dynamically changing links between individuals. Desirable partners are available, able and willing to provide benefits [21, 22]. Partners are assessed based on different cues. Generosity is often considered a reliable cue, because generous partners seem more likely to return an individual’s investment in the partnership and may even result in a competition to help the most, as a way to attract partners [22]. Other qualities may also serve as guide to assess partners. For example, players with an aversion to payoff inequities take into account the success of (potential) partners in comparison to their own performance [23, 24]. Moreover, maintaining mutually beneficial interactions is just as important as searching for and recruiting new partners. Reciprocity is one of the best studied mechanisms driving cooperation in long-lasting interactions [25–28]: *direct* reciprocity follows the principle ‘I help you and you help me’, while *indirect* reciprocity implements ‘I help you because you helped someone else’.

The models for the evolution of cooperation in structured populations generally introduce a clear distinction between structure and strategy. Individuals update their strategies—typically *cooperation* or *defection*—and, if partner choice is allowed, individuals can also adjust their links. Recently, Wardil & Hauert [29] broke with this tradition an introduced a simple theoretical framework to model dynamic social networks based on individual actions instead of interactions between individuals. An act of cooperation provides a benefit *b* at a cost *c* and can be represented by a directed link pointing from the provider to the recipient. Assuming unencumbered benefits, links can be added (or removed) without the consent of the recipient.

This apparently simple change induces a paradigm shift: the social network now reflects the actual social (inter-)actions and the neighbourhood of an individual naturally encodes its behavioural type, eliminating the dichotomy between structure and strategy. The behavioural type of each individual is characterized through its local network structure and quantified by

L=(g-l)/(g+l), (1)

where *g* denotes the individual’s generosity measured as the number of donations (recipients) and *l* indicates the number of providers. Individuals with positive *L* can be classified as altruists, those with negative *L* as egoists and an *L* close to zero indicates fair players. The density of the network indicates the degree to which the population is engaged in in cooperative activities. This framework amalgamates direct and indirect reciprocity into the concept of network reciprocity, where benefits may return to the provider either directly through bi-directional links or indirectly through longer directed cycles.

## Results

Here, we report the results of behavioural experiments where we investigate the interplay between cooperative actions and network formation following the theoretical framework introduced in [29].

### Setup

Participants played 60 rounds of a donation game (without knowing the exact number of rounds). In each round they had to chose whether and to whom they wanted to provide a benefit of two tokens at the cost of one token. Individuals were identified by unique, anonymous ID’s with access to their current payoff and generosity (number of donations). Cooperative actions are represented as directed links pointing from the donor to the recipient. The donor pays the costs and the recipient receives the benefits as long as the link exists, i.e. until the donor decides to stop providing. Each participant was allowed to adjust up to two links by removing existing ones or adding new ones. Note that participants could only choose whether and to whom to provide benefits but had no control over who provided benefits to them. Every round lasted for 30 seconds and at the end of each round the network was updated and the payoffs for that particular round determined.

**Fig 1:**
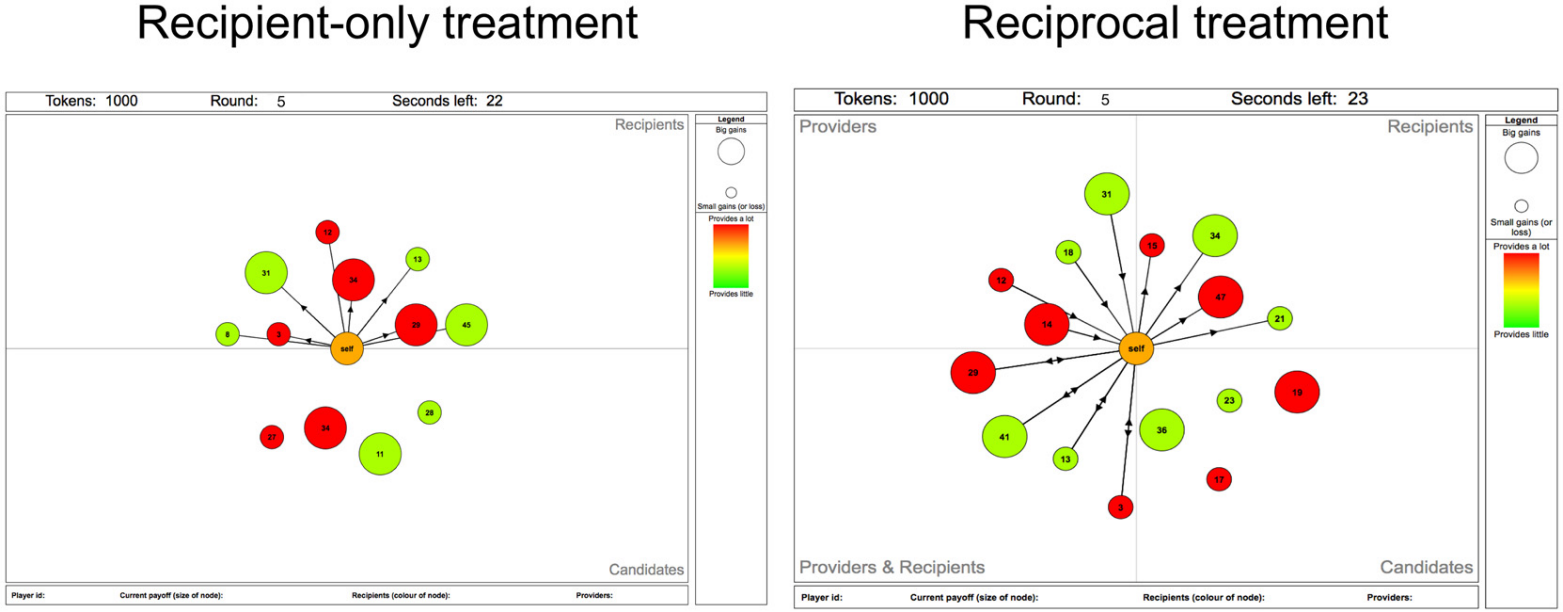
Graphical interface. *Recipient-only* is shown in (a) and the *reciprocal* treatment in (b). The focal participant is represented by the central node. Directed links point from donors to recipients. The size of the node reflects the payoff in the previous round of that individual, while the colour reflects its generosity, i.e. number of recipients entertained. The four categories of providers, recipients, candidates, and reciprocators are visually arranged the focal participant. Note that reciprocators are identified as “Providers & Recipients” to avoid framing effects. The upper bar displays the current round, the net number of tokens accumulated up to the current round, and the number of seconds left before the end of the round. By clicking on any node, the lower bar shows the ID, the current payoff, the number of providers, and the number of recipients. IDs uniquely identify other individuals but are also unique for each participant such that no information could be obtained from glancing on someone else’s screen.

To assess the effect of reciprocity, there were two treatments. In the *recipient-only treatment*, each participant saw the IDs of the *recipients* of donations as well as a random sample of *candidates*. In particular, participants could not see the IDs of their *providers* such that it was impossible to reciprocate and return benefits directly to the providers. In the *reciprocal treatment* participants additionally saw the IDs of their *providers*, which admitted opportunities for direct reciprocation. For easy identification, individuals that both received from and provided to the participant were visually grouped as *reciprocals*. The graphical interfaces for the two treatments are shown in Fig 1. Individuals participated in only one treatment. The average number of participants in each session was 30 participants.

In contrast to previous experiments, where an initial network was present, the ‘network’ starts out as a set of disconnected nodes. Thus, the first question is whether a network will indeed emerge and, if it does, to characterize its social structure. The second question then becomes what mechanisms drive the emergence of social networks. Of particular interest is the extent to which payoffs and generosity, which is defined as the number of cooperative actions, affects a participant’s decision to add or to remove links. In this regard, our conclusions complement studies on image scoring [25], inequity aversion [23], and on payoff-based update dynamics like imitate-the-best or pairwise comparison [17].

### Analysis

**Fig 2:**
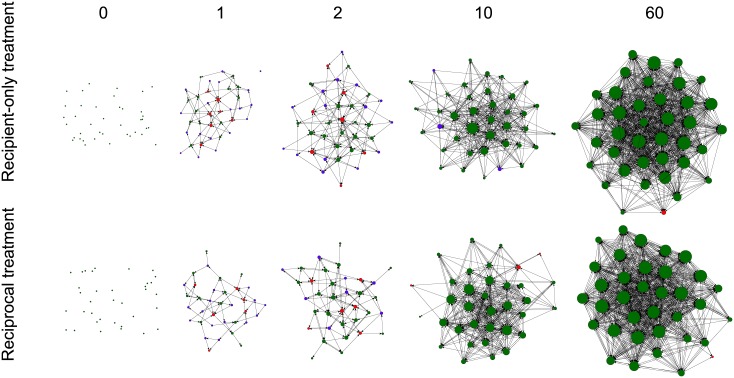
Network snapshots. The snapshots are at rounds 0, 1, 2, 10, and 60 for one session in the (a) recipient-only treatment and one session in the (b) reciprocal treatment. The size of nodes indicates an individual’s connectedness, *g*+*l*, i.e. the total number of providers (incoming links, *l*) and recipients (outgoing links, *g*), while the colour reflects its behavioural type: altruists (blue, 1/3 < *L*_*i*_ ≤ 1), fair players (green, −1/3 ≤ *L*_*i*_ ≤ 1/3) and egoists (red, −1 ≤ *L*_*i*_ < −1/3). Individual generosity, *g*, and received donations, *l*, are normalized by the session size to permit comparisons and calculations across sessions.

**Fig 3:**
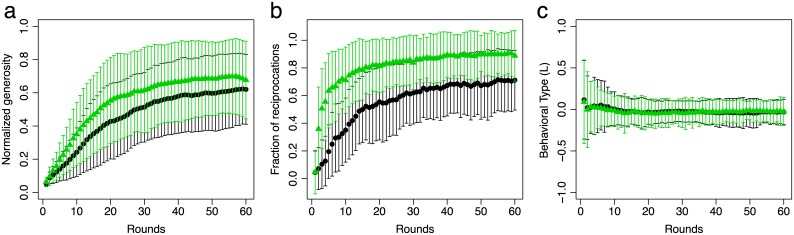
Generosity, direct reciprocation, and behavioral type. (a) The average generosity and (b) the fraction of bidirectional links, which quantifies the number of direct reciprocations, increase in the first rounds and reach an approximately stationary regime in the last rounds in both recipient-only (black, circles) and reciprocal treatment (green, triangles). The bars indicate one standard deviation. (c) The average behavioural type *L* = (*g* − *l*)/(*g*+*l*) is close to zero during all rounds. In (a) individual generosity, *g*, is normalized by the session size to permit comparisons and calculations across sessions.

Networks of cooperation readily emerge in our experiments, as illustrated by network snapshots in Fig 2. The generosity of an individual in any given round is quantified by its number of donations (or recipients), *g*, whereas the network density reflects the average generosity of all participants, see Fig 3a. In both treatments network density, or average generosity, increases during the first rounds until an approximately stationary regime with dense networks is reached. In the stationary regime, each individual provides, on average, benefits to 62% of the other participants in the recipient-only treatment and to 69% in the reciprocal treatment. This difference is not significant (*χ*^2^(1) = 3.13; *p* = 0.08). The average for the stationary regime is computed over the last five rounds but the results do not depend on this number, as long as all rounds are in the stationary regime. More specifically, stationary regime includes rounds where the absolute value of the numerical derivative of the average generosity is less than 10% of the maximum derivative (see S1 File). Note that the large variance reflects variation in individual behaviour even in the stationary regime of the network density.

Direct reciprocation is measured as the fraction of bi-directional links in the emerging networks, see Fig 3b. Not surprisingly, the fraction of direct reciprocation in the stationary phase is significantly higher, 90%, than in the recipient-only treatment with 71% (*χ*^2^(1) = 20.18; *p* < 0.001). Interestingly, however, in *both* treatments the fraction of direct reciprocation is larger than in randomly generated networks with the same number of links (recipient-only: *F*(1) = 23.76, *p* < 0.001; reciprocal *F*(1) = 283.7, *p* < 0.001). Hence, direct reciprocation is not merely a by-product of the high density of links. Note that it is not possible to intentionally return benefits to providers in the recipient-only treatment and yet direct reciprocation is larger than expected by chance. The behavioural type of individuals, Eq 1, rapidly approaches fairness (*L* ≈ 0) after a few initial rounds, see Fig 3c. Treatment does not have an effect on behavioural type (*χ*^2^(1) = 0.17; *p* = 0.68).

**Fig 4:**
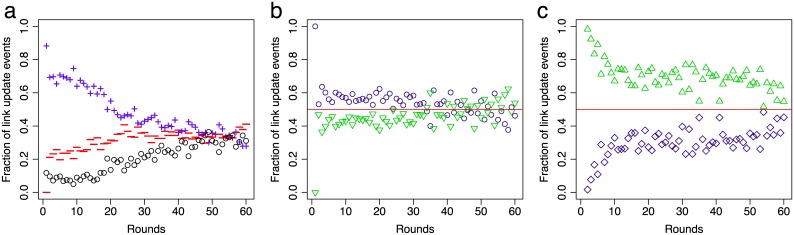
Time evolution of link update events. (a) Fraction of added links (blue, plus) and deleted links (red, minus) in the recipient-only treatment. The sum of link additions and deletions subtracted from the total number of link updates—which is two times the number of participants in the session—yields the number of renounced link updates (black, circle). Note that in the last rounds the number of added and removed links is approximately the same. Similar behaviour is observed in the reciprocal treatment (not shown in the figure). (b) In the reciprocal treatment, links are added slightly more often to candidates (blue, circle) than to providers (green, triangle), while (c) links from recipients (green, triangle) are removed more often than links to reciprocals (blue, square).

The activity of each participant can be assessed by the number of links added or removed in each round. Since each participant is allowed to make up to two changes per round, the maximum number of changes across the network is two times the number of participants. Fig 4a shows the fraction of link additions, link deletions and renounced changes, i.e. accounting for participants that decided to make no or only a single change in the respective round.

Although in all sessions stationary regimes were reached, participants remained active, adding and removing links at similar rates until the end of the experiment. Interestingly, the fraction of links added to providers in the reciprocal treatment is roughly the same as the fraction added to candidates, see Fig 4b. This suggests that participants have to find a balance between securing their cohort of providers through direct reciprocation and increasing their exposure by adding links to new nodes in the hope to attract more providers. In contrast, the fraction of links removed from recipients is significantly larger than the fraction removed from reciprocals (binomial test; *p* < 0.001), Fig 4c. Both mechanisms effectively increases the life-span of links through direct reciprocation [30].

**Fig 5:**
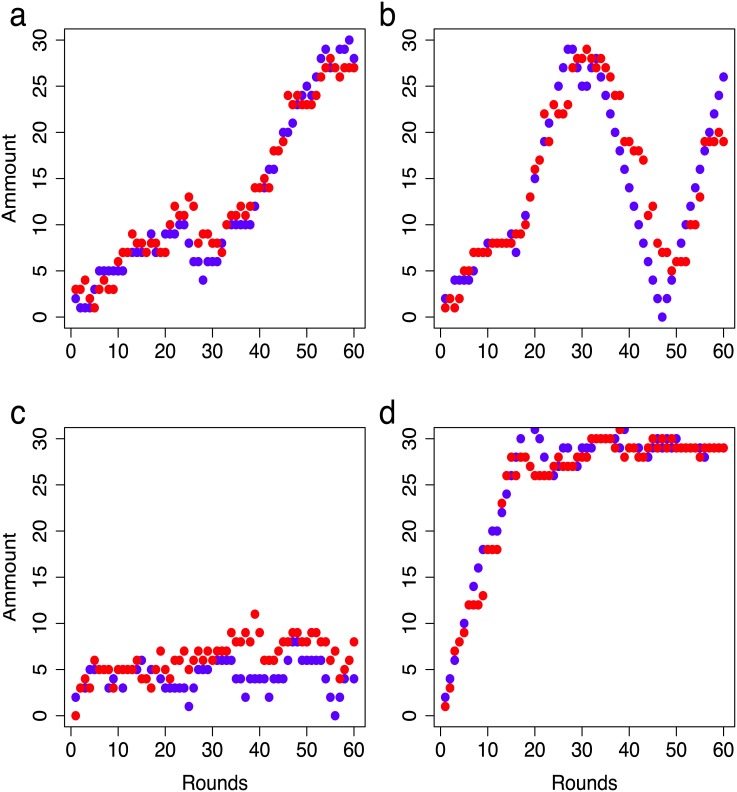
Recipients and providers. Time evolution of the number of recipients (blue) and providers (red) for selected participants from reciprocal treatment. Note the striking correlation between the numbers of providers and recipients. We show participants exhibiting four types of time evolution: (A) small variation of the number of recipients in the first half, but large variation in the second half; (B) large variation in both halves; (C) small variation in both halves; (D) large variation in the first half and small variation in the last half.

The basic pattern behind the network emergence in both treatments is simple: the number of recipients is positively correlated with the number of providers and can be measured using Kendall’s *τ* coefficient with *τ* ∈ [−1, 1]. Large *τ* indicate strong positive correlation, whereas small *τ* reflect strong negative correlation. The average *τ* in the recipient-only treatment is *τ* = 0.69 and *τ* = 0.68 in the reciprocal treatment. The strong positive correlation means that the number of recipients and providers changes in the same direction: if one is increasing (decreasing), the other one is also increasing (decreasing), see Fig 5. We did not observe any participant that succeeded in the attempt of withdrawing help in order to increase his own payoff: participants with few recipients had few providers. Conversely, participants that increased their generosity were always compensated by increasing numbers of providers. Interestingly, it is not possible to decide whether changes in generosity (recipient numbers) trigger changes in the number of providers or vice versa.

Networks emerge as consequence of individual actions. Therefore it is natural to ask what kind of information individuals are taking into account to update links. More specifically, do payoff and/or generosity of others matter when adding or removing links? To answer this question we characterize link update events, i.e. link additions and link deletions, in terms of payoff and generosity differences between the donor and recipient. In particular, it is enlightening to determine whether individuals add (or remove) links to more (or less) successful or generous individuals. An individuals payoff, *π*, is determined by its number of recipients and providers: *π* = *l* ⋅ *b* − *g* ⋅ *c*, where the benefits of a cooperative action are set to *b* = 2 and its cost to *c* = 1. The relative payoff of a model individual *m* as compared to the focal individual *f* is simply given by the payoff difference Δ*π* = *π*_*m*_ − *π*_*f*_. Analogously the relative generosity is given by Δ*g* = *g*_*m*_ − *g*_*f*_.

**Fig 6:**
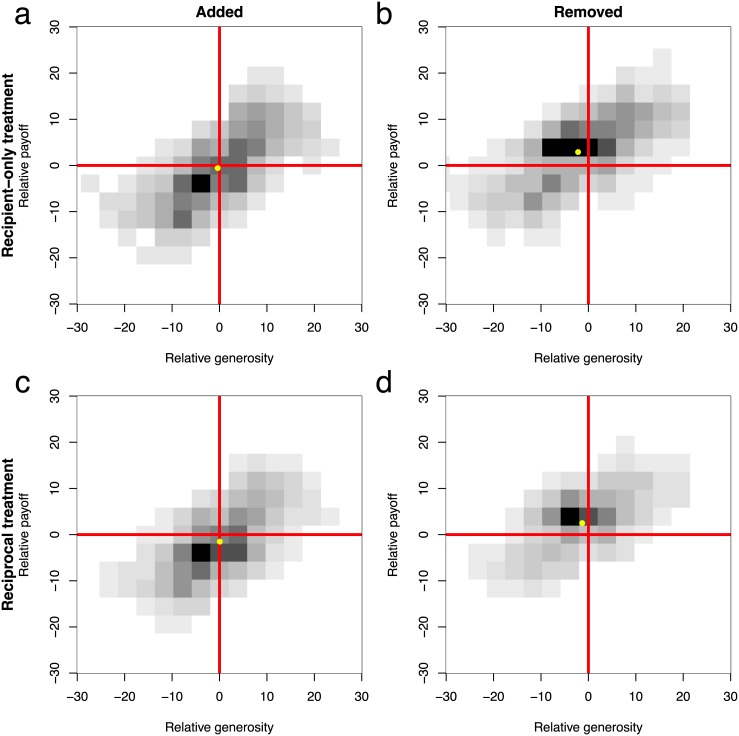
Distribution of link update events in terms of relative generosity Δ*g* and relative payoff Δ*π*. The mean $\left(\bar{\Delta g},\bar{\Delta \pi }\right)$ is shown as the yellow circle. (a) In the recipient-only treatment, most links are added to less successful targets. Generosity does not have a significant effect (51% added to less generous, *p* = 0.88). The mean is (−0.37, −0.57). (b) Links to more generous and less successful are rarely removed. Here, update events are spread throughout the other quadrants. The mean is (−2.17, 2.89). (c) In the reciprocal treatment, most links are added to less successful targets. The slightly larger fraction added to more generous is not statistically significant (52% added to less generous, *p* = 0.08). The mean is (−0.62, −1.93). (d) Links to more successful targets are removed more often. The effect of generosity depends on the target category: links to more generous reciprocals are removed more often, whereas links to less generous reciprocals are removed more often (shown in the inset panel). For reciprocators the mean is (5.36, 3.09), whereas for non-reciprocators the mean is (−6.14, 0.70).

Fig 6 shows the joint histogram *p*(Δ*g*,Δ*π*) of link update events. Note that the first 10 rounds are not taken into account because initially nodes are disconnected and hence no providers or recipients exists. The marginal distributions *p*_*g*_(Δ*g*) and *p*_*π*_(Δ*π*), indicate a clear effect of payoff differences: 60% (recipient-only) and 61% (reciprocal) were *added to less* successful targets, whereas 67% (recipient-only) and 59% (reciprocal) were *removed from more* successful targets. The effect of generosity is less clear and varied between treatments. The only significant effect applies to link deletion: 56% (recipient-only) were removed from less generous targets. In the reciprocal treatment, generosity plays different roles depending on whether the target is reciprocating or not: 73% were removed from more generous reciprocators, whereas 71% were removed from less generous non-reciprocators. All proportions are significantly different from randomly adding/removing with 50% chance (binomial test; *p* < 0.001).

The counterintuitive fact that links to more generous reciprocators are the ones most often removed in the reciprocal treatment can be understood by analyzing the joint distribution *p*(Δ*g*,Δ*π*). Table A in S1 File shows that 55% of the removed links were linked to more generous *and* more successful targets, suggesting that aversions to payoff inequity outcompetes reciprocation.

In summary, in both treatments consistent behavioural patterns emerge based on relative success: most links were added to less successful targets and most were removed from more successful ones. In contrast, relative generosity had significant effects only on link deletion. In the recipient treatment, most links were removed from less generous targets but in the reciprocal treatment: links to more generous reciprocators were removed more often, whereas links to less generous non-reciprocating recipients were the ones removed more often.

## Discussion

We have shown that dense social networks readily and spontaneously emerge based on costly cooperative actions regardless of whether direct reciprocation is possible. The emerging social structure is egalitarian in both treatments. In particular, no stratification in terms of generosity or payoffs was observed. All participants are fair players: the number of providers equals the number of recipients. An individual which provides benefits only to a few recipients attracts only a few providers. Individual behaviour resembles an indirect version of the *tit-for-tat* strategy: ‘what you do to others, others do to you’ or, conversely, ‘what others do to you, you do to others’. However, the statistics are inconclusive whether participants are attracting and loosing incoming links as a consequence of their own behaviour, or whether participants adjust their behaviour in response to the behaviour of others toward them, or a combination thereof.

In our experiment, individuals exhibit a marked aversion to payoff inequity: help was withdrawn from more successful individuals and provided to less successful ones. As a result participants tend to be fair players: adding links decreases the focal individual’s payoff, but attracts providers—whereas, removing links increases the focal individual’s payoff, but prompts the loss of providers. Hence, the number of recipients and providers tends to remain equal. In other words, aversion to payoff inequity shapes the network formation such that individuals are characterized by fairness, *L* ≈ 0. Note that fairness is often defined as self-centred inequity aversion [23]. To avoid confusion, here we use the term ‘aversion of payoff inequity’ for the motivation driving the link updates and we use the term ‘fairness’ to characterize the outcome *L* ≈ 0. Self-interest could, in principle, entice individuals to reduce helping in order to increase their payoff but because of the strong correlation between removing and loosing links this would drive a node into complete isolation, an outcome that is not observed in our experiment. Instead, our results suggest that, since the number of recipients is positively correlated with the number of providers, participants recognize that it is in their best interest to be generous (at least to a certain degree) and insofar fairness is preserved.

Individual behavioural preferences can be inferred from the most frequent type of link update of each participant. In terms of relative generosity, we can define such preferences as “I prefer to add to (more | less) generous targets” and “I prefer to remove to (more | less) generous targets”, as well as analogous statements in terms of higher and lower payoffs. For example, if for a given participant, more than half of targeted nodes are more generous, we say that this participant prefers to help more generous individuals. Interestingly, some participants display no preference, but among those participants that do, it turns out that aversion to payoff inequity is the most consistent preference across treatments. In addition, we observed that well-off and more generous reciprocators lose benefits in spite of their good standing as generous players, as opposed to well-off and more generous non-reciprocating recipients. The analysis of individual preferences based on link update events yields the same qualitative results (S1 File).

Intriguingly, high levels of generosity emerge even if direct reciprocation is not an option. Aversion to payoff inequity was observed in both treatments, whereas reciprocity towards generous players was not consistently observed. Aversion to payoff inequity seems to outcompete reciprocity considerations: in the reciprocal treatment links to more successful and more generous reciprocators were likely removed! It is as if participants were confident that successful and cooperative reciprocators would be less likely to retaliate if a link to them is removed. In the recipient-only treatment, although participants could not intentionally reciprocate, the number of bi-directional links in the stationary regime was larger than what would be expected in a random network with the same number of links. The reason is that aversion to payoff inequity gives rise to a tendency of mutual help between participants with similar payoff, a bias that is not present in the equivalent random network.

The choice between adding links to new candidates or to providers in the reciprocal treatment gives rise to a second dilemma: is it better to secure providers or to increase the exposure in an attempt to attract new ones? In our experiment participants could make up to two link updates per round and, in general, opted to increase exposure slightly more often than to secure providers, see Fig 4. This dilemma would be more pronounced if participants were allowed to update only one link per round. On the other side, if there were no restrictions in the number of updates per round, participants could reciprocate and still increase their exposure.

Theoretical models demonstrate that social networks based on cooperative actions readily and spontaneously emerge [29]. Cooperative behaviour is transmitted through imitation of more successful models: links are preferentially added to more successful and more cooperative individuals while removed from more successful but less cooperative ones. Our experiments confirm the ready emergence of social networks but, in contrast to theory, participants do not add links to more successful individuals, but actually to less successful ones. Although update rules based on imitation of more successful strategies have been widely adopted in theoretical studies, behavioural experiments, ours included, fail to provide support for merit-based update rules like imitate-the-best or pairwise comparisons [17]. Instead, inequity aversion and fairness considerations appear to be of paramount importance in empirical settings, which are not restricted to human interactions [31]. Theoretical work on cooperative actions [29] inspired our experiments and now our experiments may, in turn, help inspire theoretical investigations into imitation processes and the evolution of inequity aversion, in particular.

In conclusion, our behavioural experiments empirically investigate the dynamics of social networks based on cooperative actions and demonstrate that dense networks of cooperation readily emerge, even if direct reciprocation is not possible. Individuals are driven by an aversion to payoff inequities as evidenced by behavioural patterns, which redirect benefits from those that have more to those that have less than the individuals themselves. Such individual behaviour triggers the emergence of highly egalitarian network structures. Interestingly, if direct reciprocation is possible, the network structure essentially remains unchanged but generous and well-off reciprocators are more likely to loose their beneficiaries than well-off but selfish reciprocators. While this may seem counter-intuitive at first, it reflects the need to balance costly cooperation and potential benefits of increased exposure. The focus on cooperative actions as the building blocks of (human) social interactions, helps to reveal basic behavioural patterns that control the structure and dynamics of social networks.

## Methods

### Ethics statement

All participants in the experiment provided informed consent by checking a box on a welcome page prior to the start of the experiment. Participants could only proceed to the experiment after consent was provided. Screenshots of the consent process are available in S1 File. The research was approved by the University of British Columbia Behavioural Research Ethics Board under reference H12-02710.

### Experiment setup

We conducted behavioural experiments with 276 human subjects recruited from first and second-year science students at The University of British Columbia. Participants were divided into 9 sessions (30 participants in each session, on average) and interacted with others in the same session through a graphical interface. Participants were represented as nodes and the cooperative actions as directed links pointing from the donor to the recipient. Each participant was assigned a fixed but random ID. Note that a different set of random numbers was used for each participant so that no information could be learned from peeking on another screen.

There were two treatments, see Fig 1. In both treatments the focal participant was represented as the central node. In the recipient-only treatment, the recipients of the focal participant and a random sample of candidates were displayed around the central node. In the reciprocal treatment, the recipients, a random sample of candidates *and* the providers of the focal were displayed. Clicking on any node provided more detailed information: the number of recipients (also graphically displayed as a colour gradient), the number of providers, and the payoff, 2*l* − *g* (also graphically displayed as node size). We had 4 sessions in the recipient-only treatment (number of participants in each session: 31, 31, 35, and 39) and 5 in the reciprocal treatment (number of participants in each session: 17, 22, 32, 34, and 35). Note that one session of recipient-only treatment had to be cancelled due to technical problems, which prevented access to the Internet. Participants were physically present in a classroom but interacted anonymously through a custom web-based application.

Participants were initially given 1000 tokens. This amount was set to avoid bankruptcy. In each round, they had 30 seconds to decide whether or not to add or remove up to two links—a directed link represents the action of providing two tokens at the cost of one token. In the first round the only available action is to add links to candidates. A new, random sample of candidates was provided in every round. At the end of each round the entire network is simultaneously updated to reflect the decisions of all participants in the current session. To illustrate the update process, let us consider an hypothetical case in the recipient-only treatment. Suppose in the first round a participant *x* decides to add 2 links and 4 participants decide to add a link to *x*. In the second round participant *x* starts with 4 providers and 2 recipients. Since in the first round participant *x* incurs a cost of 2 tokens and receives a benefit of 8 tokens, participant *x* starts the second round with 1006 tokens. The focal participant did not know the accumulated number of tokens of other participants. Note that, in the recipient-only treatment, providers are not displayed in the graphical interface. The game ended after 60 rounds, but to avoid end-game effects participants were told that the game would last on average 100 rounds.

Losses and earnings in all rounds were added to the initial endowment and converted to Canadian dollars at the end of the game. Conversion rate was defined in a way to ensure that the advertised maximum payout of $25 was achievable regardless of the session size. The average payout was $11.90 in the recipient-only treatment and $10.42 in the reciprocal treatment. Payments were sent via email in the form of Amazon gift cards. Before the experiment started, participants had to complete a tutorial that explained the experiment as well as the graphical features of the front-end. For details on the experimental setup and screenshots of the tutorial see S1 File.

### Statistical analysis

For the statistical analysis we used linear mixed-effect models to account for within-groups and between-groups variance in the analysis of treatment effects on the average generosity, behavioural type, and fraction of bidirectional links. We used ANOVA to compare the linear mixed-effect models

$$\begin{array}{l} y_{ij}=\beta_{0}+\mu_{j}+\epsilon_{ij}\\ y_{ij}=\beta_{0}+\beta_{1}\cdot x_{ij}+\mu_{j}+\epsilon_{ij}, \end{array}$$

where *i* stands for participant and *j* for session. The variable *x*_*ij*_ is equal to one if session *j* is under treatment the reciprocal treatment and equal to zero otherwise. The variable *μ*_*j*_ introduces random effects and *ϵ*_*ij*_ stands for the residual error. The dependent variable *y*_*ij*_ was averaged over the last 5 rounds. We didn’t include the rounds in the regression because the time to reach the stationary regime is different for sessions with different size. Hence, we compare averages only in the stationary regime. The statistical analysis was done using the R package *lmer*.

For the analysis of link update events, we first built the histogram *p*(Δ*g*,Δ*π*) and then we calculated the marginal number of events *p*_*g*_(Δ*g*) = ∑_Δ*π*_ *p*(Δ*g*,Δ*π*) and *p*_*π*_(Δ*π*) = ∑_Δ*g*_ *p*(Δ*g*,Δ*π*). Let $N_{g}^{-}=\sum_{\Delta g<0}p_{g}\left(\Delta g\right)$ and $N_{g}^{+}=\sum_{\Delta g>0}p_{g}\left(\Delta g\right)$. $N_{g}^{-}$ ($N_{g}^{+}$) is the number of link updates where the target is less (more) generous than the focal. To see which kind of target is the most preferred, we tested against the null hypothesis that the proportion $N_{g}^{-}/\left(N_{g}^{-}+N_{g}^{+}\right)$ is equal to 0.5 using the binomial test (binom.test in the R package *stats*). The analogous analysis was done for the marginal payoffs, *p*_*π*_(Δ*π*). The number of events characterized by Δ*g* = 0 and Δ*π* = 0 is insufficient to support statistical analysis. We also used the binomial test to compare the number of links added to recipients and unlinked nodes against the null hypothesis of equal proportions, as well as to compare the number of links removed from reciprocals and recipients.

In order to control for any color based bias, we used two different colour schemes to represent individual generosity. In one scheme red indicates high generosity and green low generosity, whereas in the other scheme colours are switched. To check for color bias, we determined the number of red and green targets when adding and removing links. Without bias the numbers should not depend on the colors and indeed proportions do not significantly differ from 50% (binomial test; *p* < 0.001), for both added and deleted links.

## Supporting Information

S1 File Tutorial and supporting analysis. (PDF)

S1 Dataset Anonymized dataset of experiments. The S1 Dataset is a zip file archive containing anonymized data of each session in separate sub-files. Sessions in the recipient-only treatment are in the subfiles B, C, D, and E and sessions in the reciprocal treatment are in the sub-files F, G, H, I, and J. The sub-file A describes data format.
(ZIP)

## References

[1] Borgatti SP, Mehra A, Brass DJ, Labianca G. Network analysis in the social sciences. Science. 2009; 323:892–895. 10.1126/science.1165821 19213908

[2] Maynard Smith J, Szathmáry E. The Major Transitions in Evolution. Oxford: W. H. Freeman; 1995.

[3] Sigmund K. The calculus of selfishness. Princeton Univ. Press; 2010.

[4] Ohtsuki H, Hauert C, Lieberman E, Nowak MA. A simple rule for the evolution of cooperation on graphs. Nature. 2006; 441:502–505. 10.1038/nature04605 16724065 PMC2430087

[5] Nowak MA. Five rules for the Evolution of Cooperation. Science. 2006; 314:1560–1563. 10.1126/science.1133755 17158317 PMC3279745

[6] Nowak MA, May RM. Evolutionary games and spatial chaos. Nature. 1992; 359:826–829. 10.1038/359826a0

[7] Hauert C. Fundamental clusters in spatial 2 × 2 games. P Roy Soc B-Biol Sci. 2001; 268:761–769. 10.1098/rspb.2000.1424 PMC1088667 11321066

[8] Fehr E, Fischbacher U. The nature of human altruism. Nature. 2003; 425:785–791. 10.1038/nature02043 14574401

[9] Bala V, Goyal S. A noncooperative model of network formation. Econometrica. 2000; 68:1181–1229. 10.1111/1468-0262.00155

[10] Pacheco JM, Traulsen A, Nowak MA. Coevolution of strategy and structure in complex networks with dynamical linking. Phys Rev Lett. 2006; 97:258103 10.1103/PhysRevLett.97.258103 17280398 PMC2430061

[11] Perc M, Szolnoki A. Coevolutionary games—A mini review. BioScience. 2010; 99:109–125. 10.1016/j.biosystems.2009.10.003 19837129

[12] Grujic J, Fosco C, Araujo L, Cuesta JA, Sanchez A. Social Experiments in the Mesoscale: Humans Playing a Spatial Prisoner’s Dilemma. PLOS ONE. 2010; 5:e13749 10.1371/journal.pone.0013749 21103058 PMC2980480

[13] Traulsen A, Semmann D, Sommerfeld RD, Krambeck HJ, Milinski M. Human strategy updating in evolutionary games. Proc Natl Acad Sci USA. 2010; 7:2962–2966. 10.1073/pnas.0912515107 PMC2840356 20142470

[14] Gracia-Lazaro C, Ferrer A, Ruiz G, Tarancon A, Cuesta JA, Sanchez A, Moreno Y. Heterogeneous networks do not promote cooperation when humans play a Prisoner’s Dilemma. Proc Natl Acad Sci USA. 2012; 109:12922–12926. 10.1073/pnas.1206681109 22773811 PMC3420198

[15] Rand DG, Nowak MA, Fowler JH, Christakis NA. Static network structure can stabilize human cooperation. Proc Natl Acad Sci USA. 2014; 111:17093–17098. 10.1073/pnas.1400406111 25404308 PMC4260616

[16] Szabo G, Fath G. Evolutionary games on graphs. Phys Rep. 2007; 446:97–216. 10.1016/j.physrep.2007.04.004

[17] Grujic J, Gracia-Lazaro C, Milinski M, Semmann D, Traulsen A, Cuesta J, Moreno Y, Sanchez A. A comparative analysis of spatial Prisoner’s Dilemma experiments: Conditional cooperation and payoff irrelevance. Sci Rep. 2014; 4:4615 10.1038/srep04615 24722557 PMC3983604

[18] Rand DG, and Arbesman S, Christakis NA. Dynamic social networks promote cooperation in experiments with humans. Proc Natl Acad Sci USA. 2011 108:19193–19198. 10.1073/pnas.1108243108 22084103 PMC3228461

[19] Wang J, Suri S, Watts DJ. Cooperation and assortativity with dynamic partner updating Proc Natl Acad Sci USA. 2012; 109:14363–14368. 10.1073/pnas.1120867109 22904193 PMC3437882

[20] Shirado H, Fu F, Fowler JH, Christakis NA. Quality versus quantity of social ties in experimental cooperative networks. Nature Commun. 2013; 4:2814 10.1038/ncomms3814 24226079 PMC3868237

[21] Baumard N, Andre Jean-Baptiste, Sperber D. A mutualistic approach to morality: The evolution of fairness by partner choice. Behav Brain Sci. 2013; 36:59–78 10.1017/S0140525X11002202 23445574

[22] Barclay P Strategies for cooperation in biological markets, especially for humans Evol Hum Behav. 2013; 34:164–175. 10.1016/j.evolhumbehav.2013.02.002

[23] Fehr E, Schmidt K. A theory of fairness, competition, and cooperation Q. J. Econ. 1999; 114:817–868. 10.1162/003355399556151

[24] Nishi A, Shirado H, Rand DG, Christakis NA Inequality and visibility of wealth in experimental social networks Nautre. 2015; 426–429. 10.1038/nature15392 26352469

[25] Nowak MA, Sigmund K. Evolution of indirect reciprocity by image scoring. Nature. 1998; 393:573–577. 10.1038/31225 9634232

[26] Trivers RL. The evolution of reciprocal altruism. Q Rev Biol. 1971; 46:35–57. 10.1086/406755

[27] Alexander R. The Biology of Moral Systems. New York: Aldine de Gruyter; 1987.

[28] Boyd R, Richerson PJ. The evolution of indirect reciprocity. Soc Networks. 1989; 11:213–236. 10.1016/0378-8733(89)90003-8

[29] Wardil L, Hauert C. Origin and Structure of Dynamic Cooperative Networks. Sci Rep. 2014; 4:5725 10.1038/srep05725 25030202 PMC4101522

[30] Taylor C, Nowak MA. Evolutionary game dynamics with non-uniform interaction rates. Theor Popul Biol. 2006; 69:243–252. 10.1016/j.tpb.2005.06.009 16427669 PMC2880897

[31] Sarah F. Brosnan S. F., de Waal F. B. M. Evolution of responses to (un)fairness. Science. 2014; 346 (6207):1251776 10.1126/science.1251776 25324394 PMC4451566

