# Supplementary material for: Targeted Cooperative Actions Shape Social Networks
Source: PLoS One. 2016 Jan 29;11(1):e0147850. doi: 10.1371/journal.pone.0147850 (PMC4733057; doi:10.1371/journal.pone.0147850)
Supplement: S1 File — (PDF) [file pone.0147850.s001.pdf]

SUPPLEMENTARY MATERIAL
Cooperative actions shape social networks

Lucas Wardil & Christoph Hauert

Department of Mathematics, University of British Columbia
1984 Mathematics Road, Vancouver B.C., Canada V6T 1Z2

Contents

A Recruitment **1**

B Consent **5**

C Tutorial **8**

D Stationary regime **23**

E Links removed from reciprocals **26**

F Individual preference analysis **26**

A Recruitment

We sent email advertising the experiment to all first and second year science students at the University of British Columbia (UBC):

> Subject: Call for participation in game theoretical experiments
>
> Dear students,
>
> Are you interested in participating in a fun experiment involving game theory? You can even earn some quick and easy money!
>
> From March 24th to March 27th Christoph Hauert and Lucas Wardil are running a series of behavioural experiments in groups up to 50 students. If you participate, you can get paid up to 25 for one hour or less. Simply go to http://lorax.math.ubc.ca/EvoLudoLabs/ and register for one of the available sessions!
>
> If you decide to register, you commit to show up at the time and place specified for your choice of session. A reminder email will be sent one day in advance.
>
> Hope to see you soon, Christoph Hauert & Lucas Wardil
>
> P.S. 1) The experiment is played on a Web interface. You should bring your own laptop or tablet. A limited number of laptops are available upon request on the registration form.
>
> P.S. 2) Note that we do NOT recruit through email.

By visiting the website mentioned in the email, participants found more information about the experiment, the time and location of the sessions, and the current number of registered participants in each session. If they wanted to participate in the experiment, they had to select one session to register for. A maximum of 50 participants for each session was set because of operational constraints. Screenshots of the registration process are shown in figures A, B, C, and D.

Participants showed up at the scheduled hour and location bringing their own laptop. If requested, laptops were provided. At the schedule time, we provided the access code for the session so participant could log in the web app.

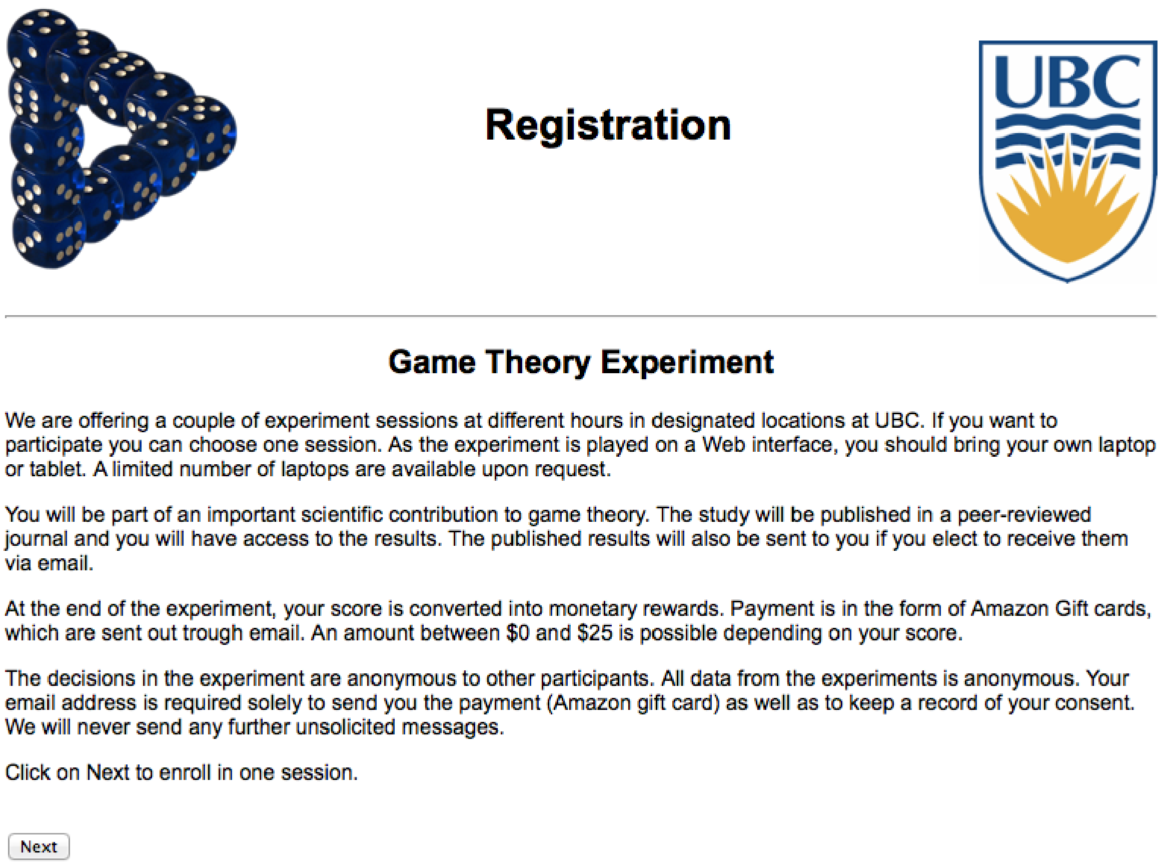

Figure A: Registration 1/4

Registration

Contact information

Please fill in your information with your personal email. Note that a valid email is essential because your payment is sent to the email that you provide.

First Name:

Last Name:

Email Address:

☐ I will need a laptop. (Note that the number of laptops is limited. Therefore we cannot guarantee the availability of sufficient numbers of laptops but we will do our best to honour all requests.)

If you bring your own laptop, please make sure that it is fully charged.

Register

Note: you can return here to change (or cancel) your registration. However, you can register only for a single session.

Figure B: Registration 2/4

Registration

Please choose a session

Choice	Date	Session	Registered Players	Maximum Players	Minimum Players
○	Tuesday, Jun 16, 2015 02:00 PM	Session 1: MATH 209	0	50	1
○	Tuesday, Jun 16, 2015 04:00 PM	Session 2: MATH 101	0	50	1

Note 1: session name refers to the actual location of the session. For example, "Session 8: MATX 1100" means that this session takes place in Mathematics Annex room 1100.

Note 2: if a session has reached the maximum number of players it will no longer be shown on this list.

Register

Figure C: Registration 3/4. This screenshot is just a sample. In the experiment participants had to select one session out of 10 options.

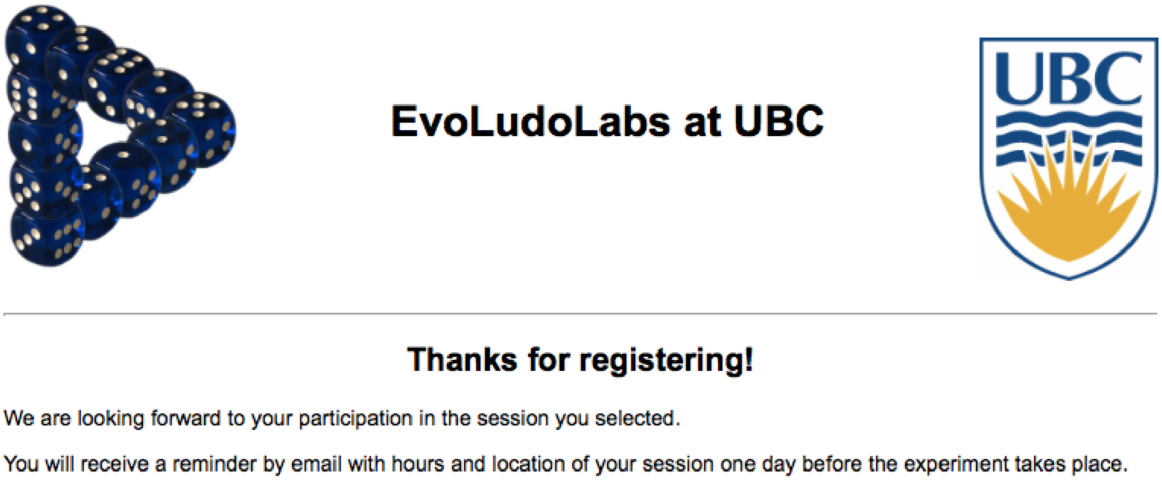

Figure D: Registration 4/4

B Consent

The consent was split into 3 pages. Participants started the tutorial only after consent was provided. Figures E, F, and G shows the informed consent webpages.

Consent Form

Game Theory Experiment

Principal Investigator

Christoph Hauert Ph.D., Mathematics Deptartment, UBC (christoph.hauert@math.ubc.ca)

Co-Investigator

Lucas Wardil Ph.D., Mathematics Deptartment, UBC (wardil@math.ubc.ca)

Potential Risks

There is no foreseeable risk associated with the experiment.

Potential benefits

You will be part of an important scientific contribution to game theory. The study will be published in a peer-reviewed journal and you will have access to the results. The published results will also be sent to you if you elect to receive them via email.

Click Next to continue.

---

page 1 of 3

Figure E: Consent 1/3

Consent Form

Summary of Experiment

Setup

All participants start with the same amount of tokens. The experiment consists of several rounds, during which you may decide to pay 1 token in order to provide 2 tokens to one or several other participants of your choice.

Procedure

Each round is 30 seconds during which time you have an opportunity adjust to whom you are providing tokens by adding new recipients and/or by removing existing recipients.

Accounting

At the end of each round, your updates are recorded and 2 tokens are added to your total score from each participant that chose to provide tokens to you while 1 token is subtracted for every participant that you chose to provide tokens to.

End

The total number of rounds is probabilistically chosen with an average of 100 rounds. Your total score is converted into monetary rewards.

Click Next to continue.

Note: detailed information about the experiment follows in an interactive tutorial.

page 2 of 3

Figure F: Consent 2/3

Consent Form

Compensation & Confidentiality

Compensation

At the end of the experiment, your tokens are converted into monetary rewards. Payment is in the form of Amazon Gift cards, which are sent out through email. An amount between $0 and $25 is possible depending on your score.

Confidentiality

The decisions in the experiment are anonymous to other participants. All data from the experiments is anonymous. Your email address is required solely to send you the payment (Amazon gift card) as well as to keep a record of your consent. We will never send any further unsolicited messages.

Consent

Taking part in this study is entirely up to you. You have the right to refuse to participate in this study. If you decide to take part, you may choose to pull out of the study at any time without giving a reason and without any negative impact on your class standing.

- [ ] Check if you would like to be contacted with the results of this experiment.
- [ ] Check to indicate that you consent to participate in the experiment.

You must consent before you can proceed to the tutorial.

Important: Take your time to carefully complete the tutorial - do not rush through, you have ample time (at least 10 minutes).

page 3 of 3

Figure G: Consent 3/3

C Tutorial

The tutorial described the graphical interface and the rules of the game. They advanced in the tutorial only if correct answers to comprehension questions were provided. Screenshots of the tutorial are shown in figures H-U.

At the end of the tutorial, participants played one round in a predefined setting where they were exposed to all combinations of relative payoffs and relative generosity, Fig. V.

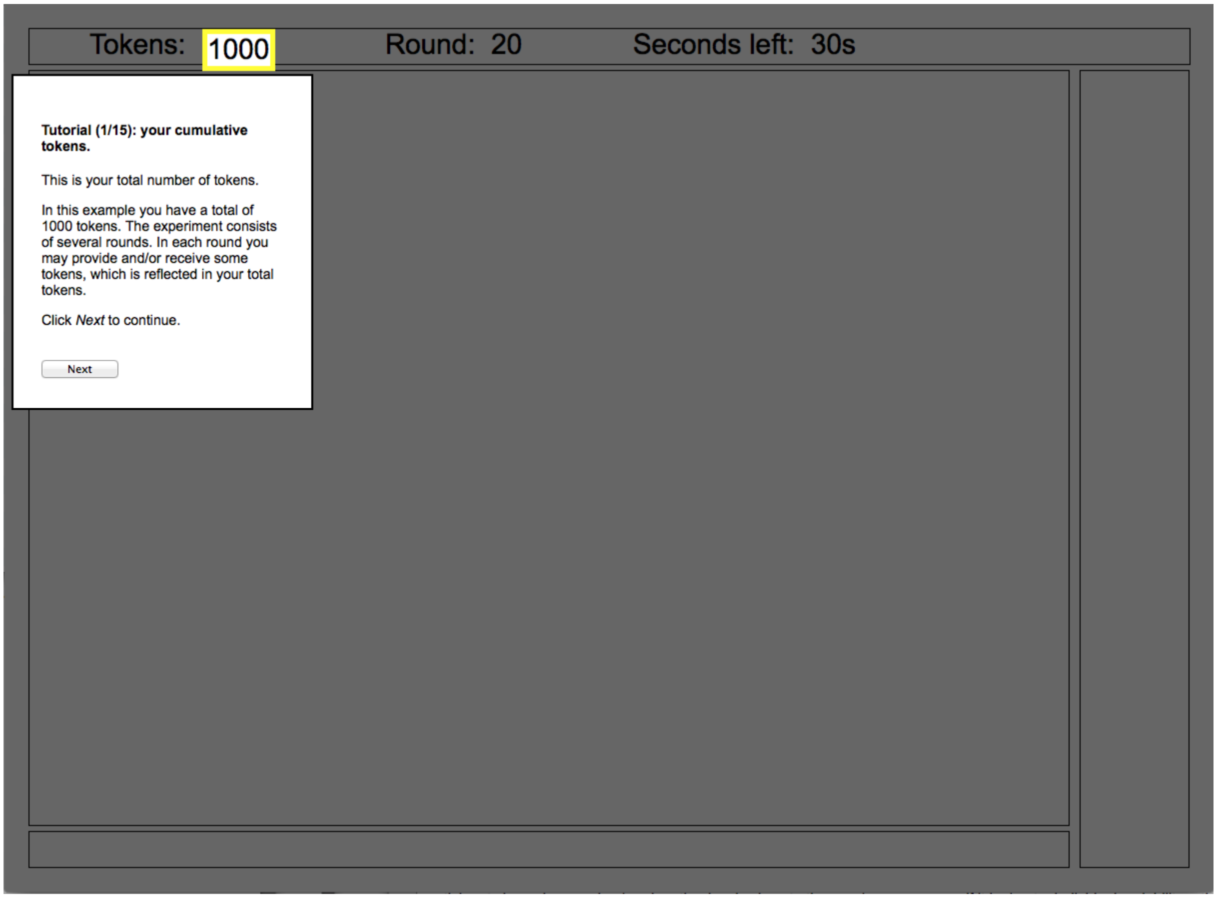

Figure H: Tutorial 1/15

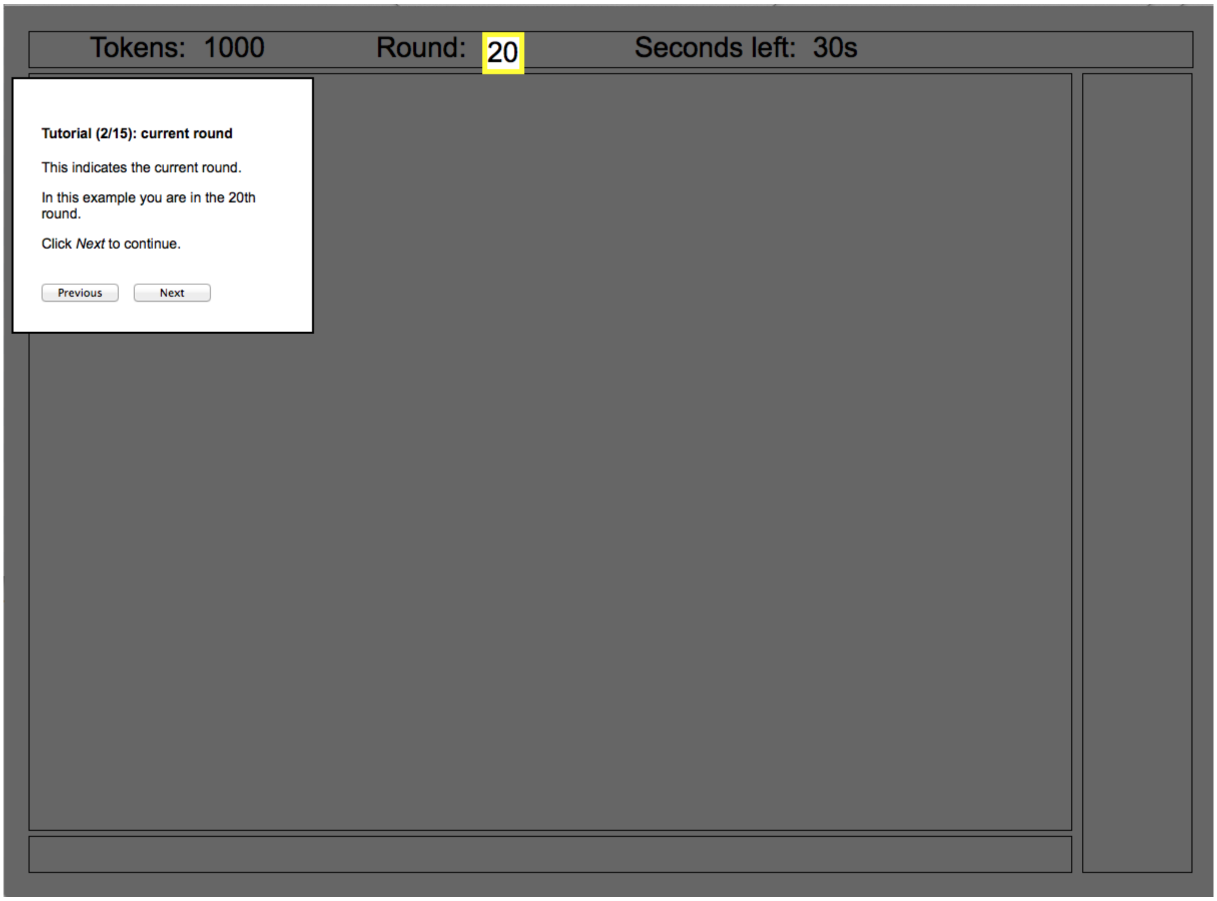

Figure I: Tutorial 2/15

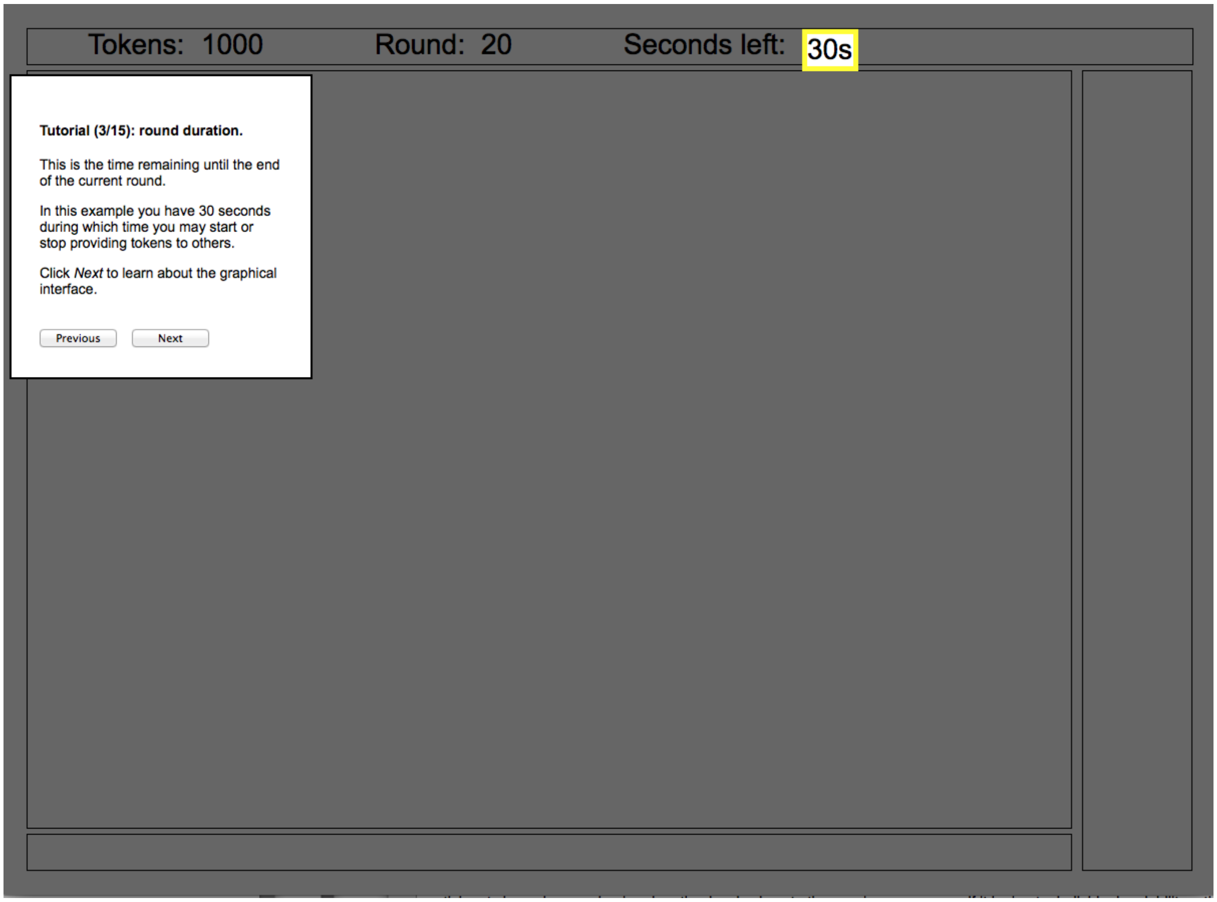

Figure J: Tutorial 3/15

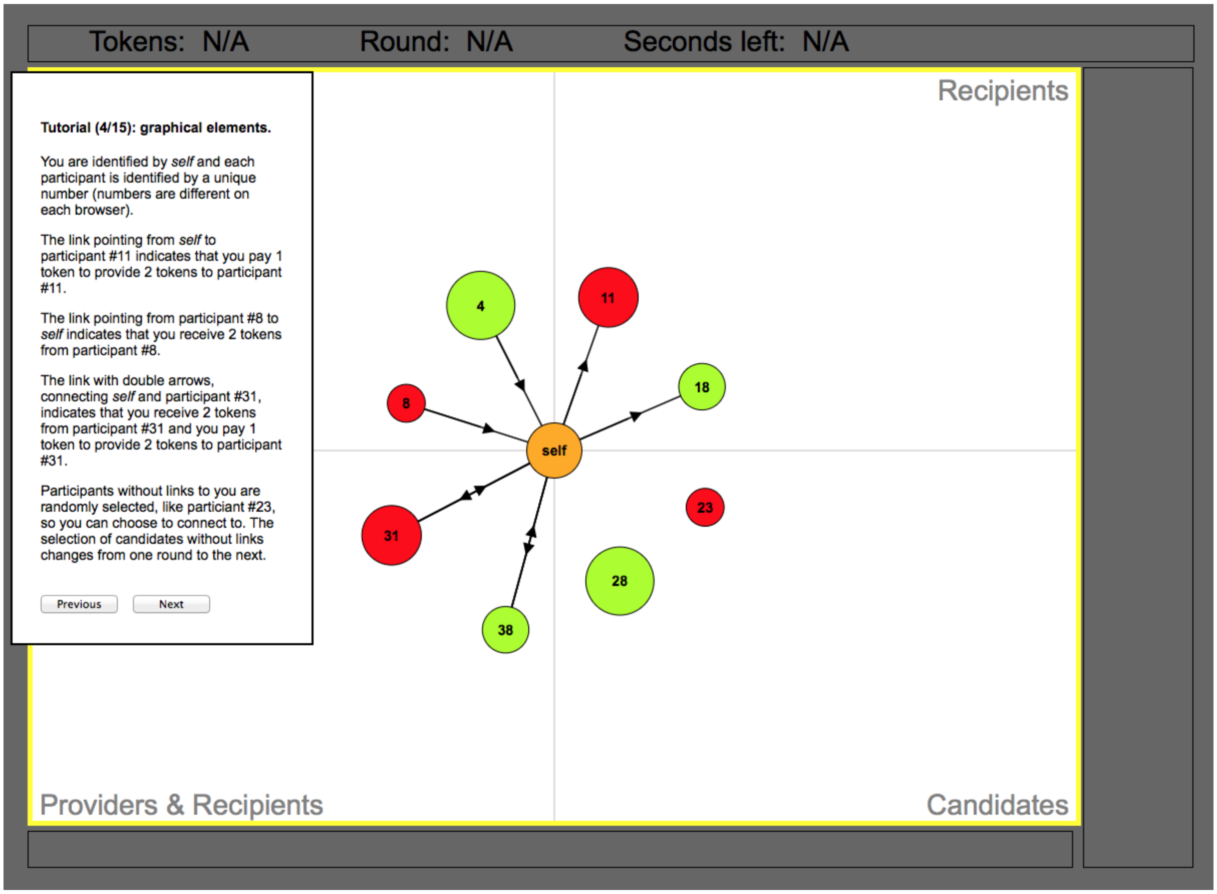

Figure K: Tutorial 4/15

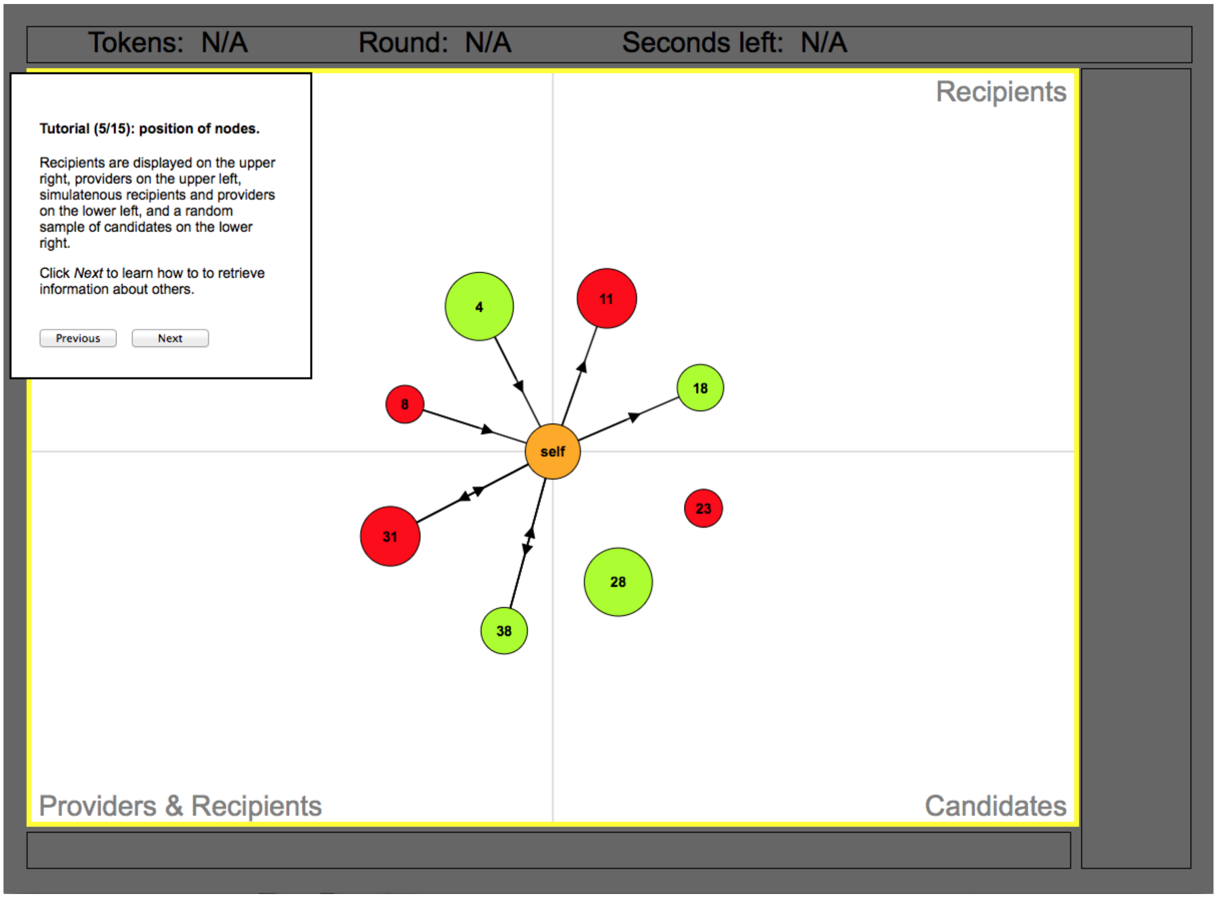

Figure L: Tutorial 5/15

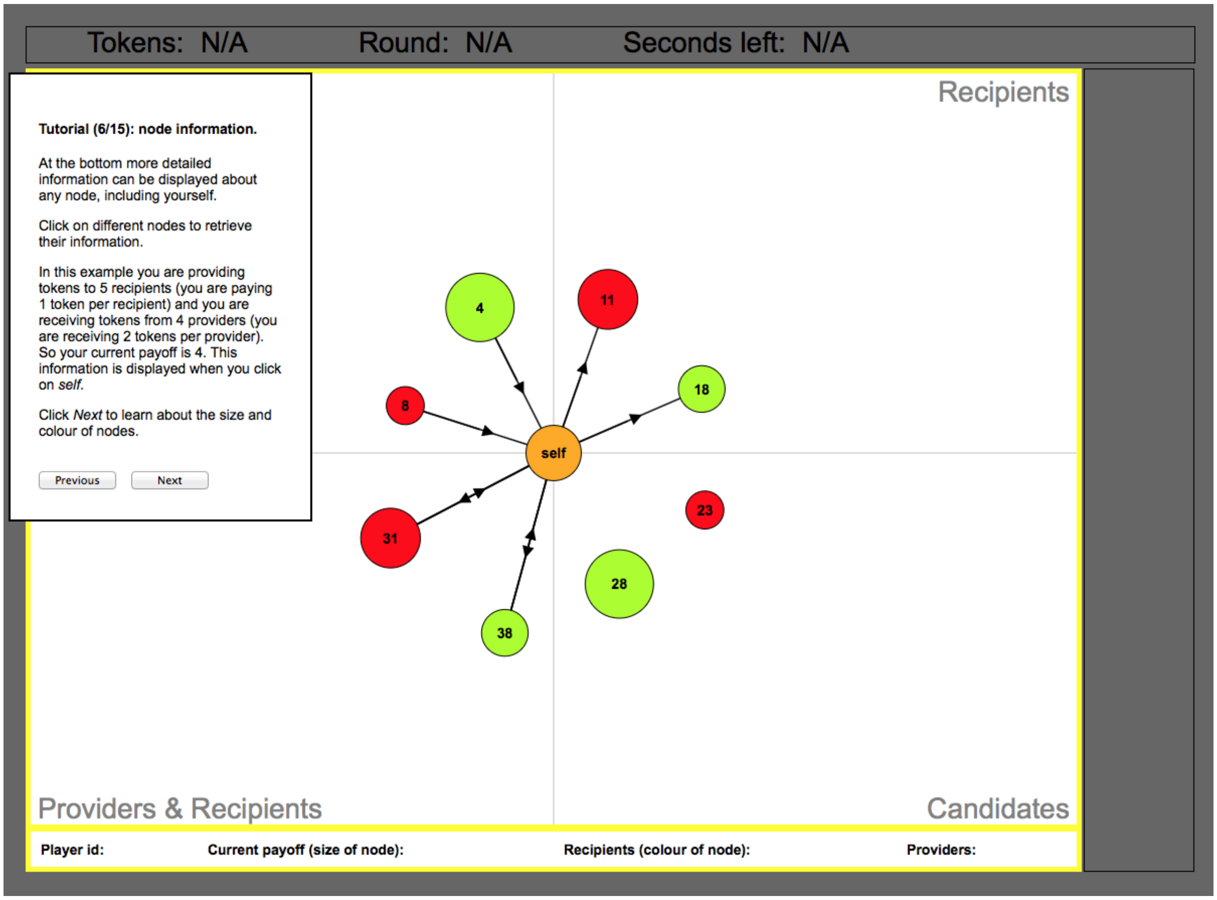

Figure M: Tutorial 6/15

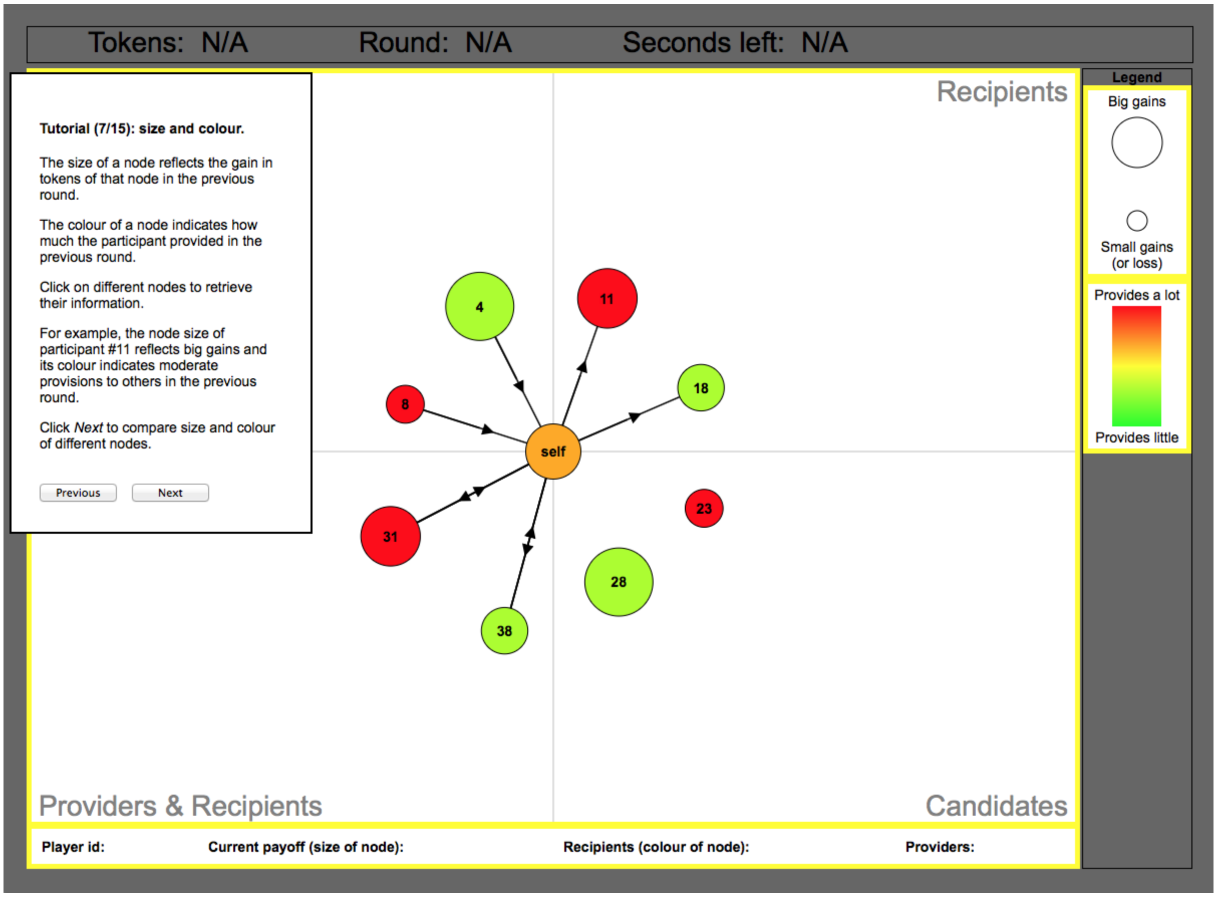

Figure N: Tutorial 7/15

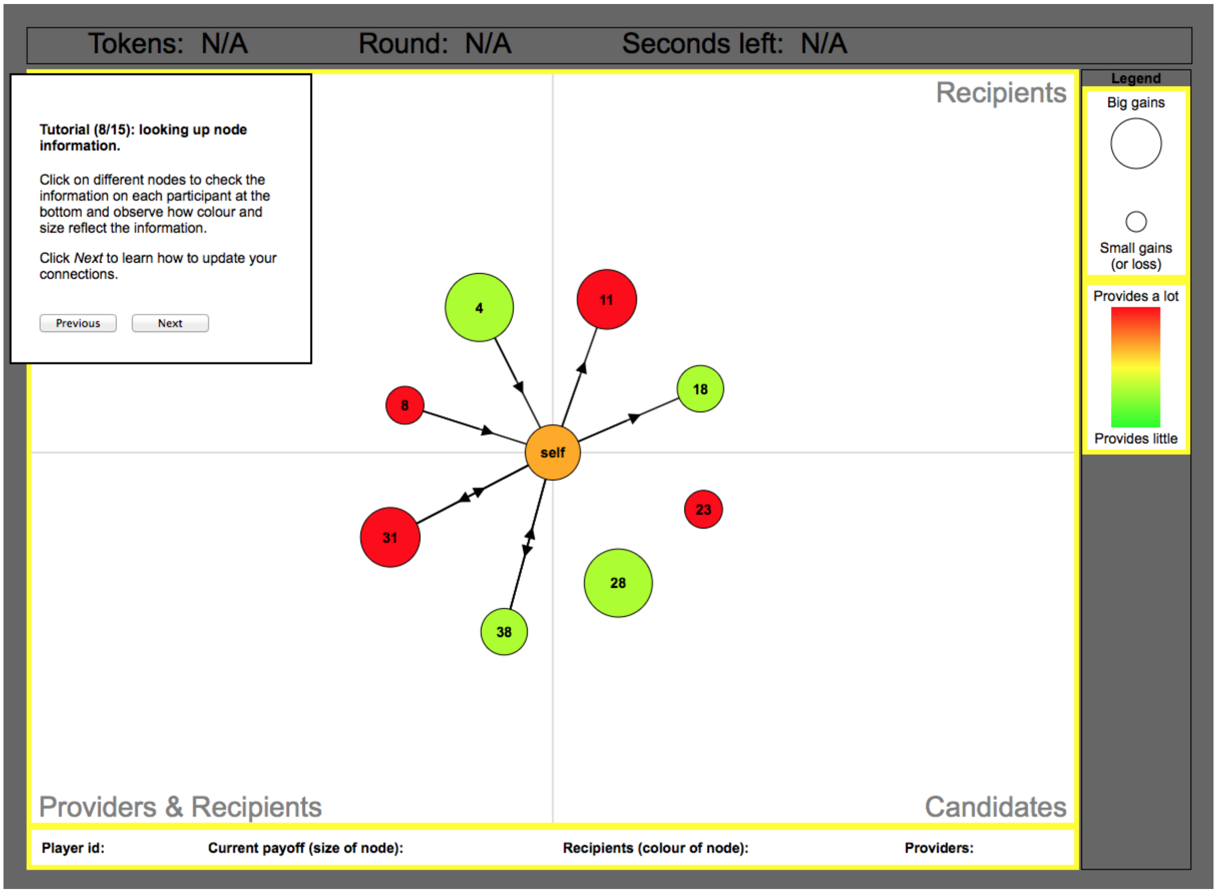

Figure O: Tutorial 8/15

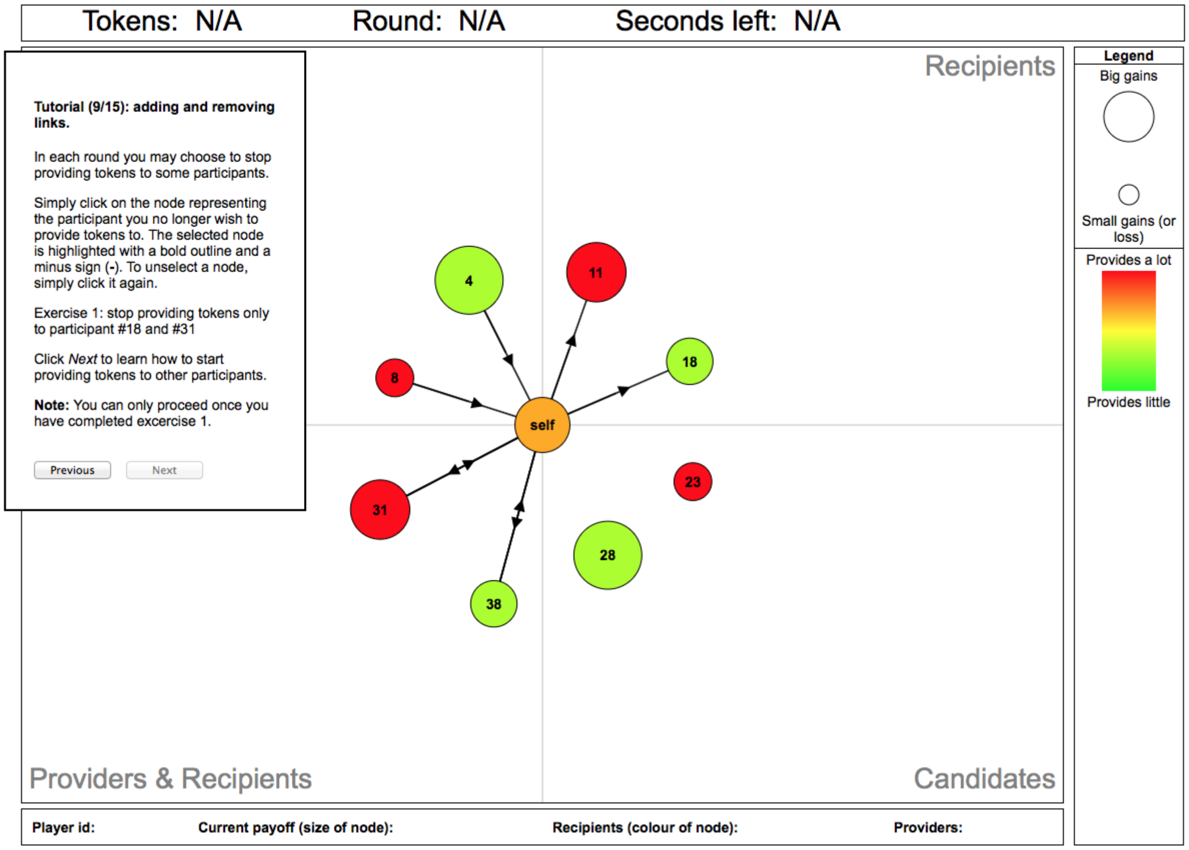

Figure P: Tutorial 9/15

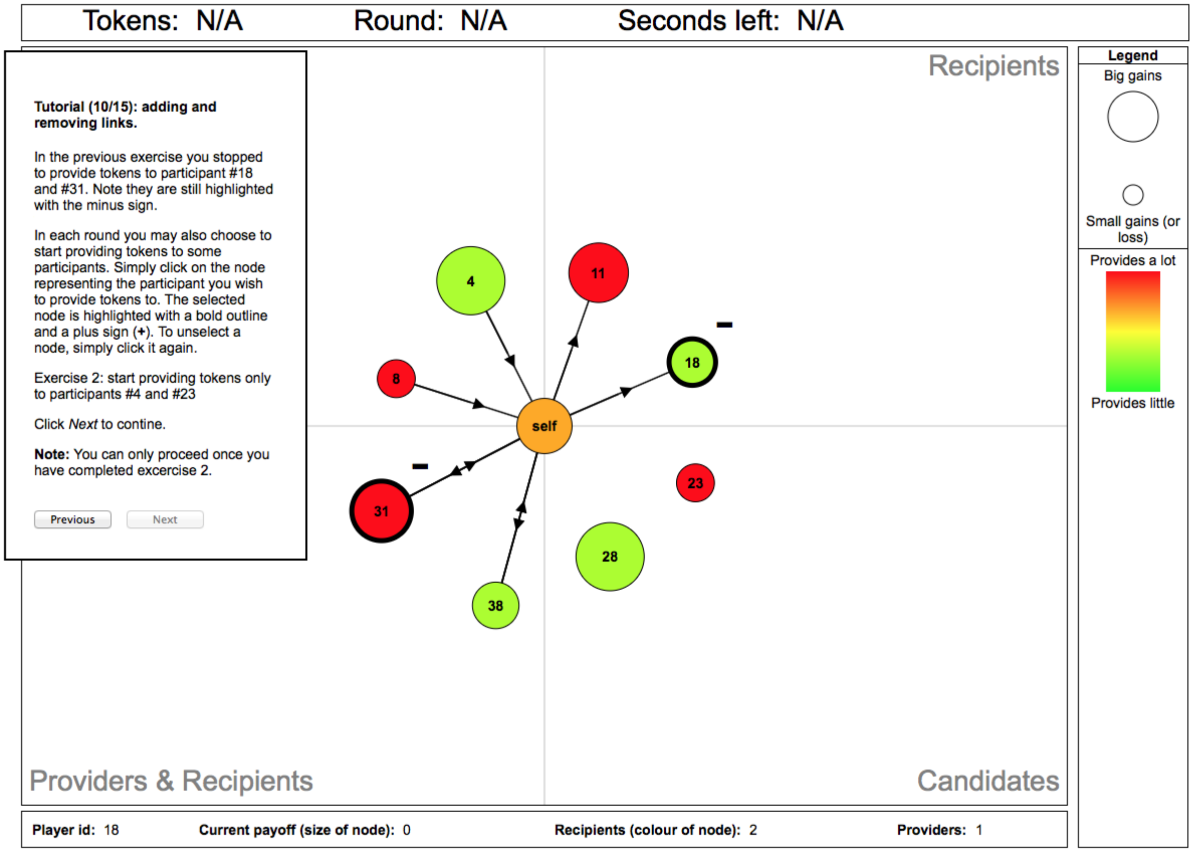

Figure Q: Tutorial 10/15

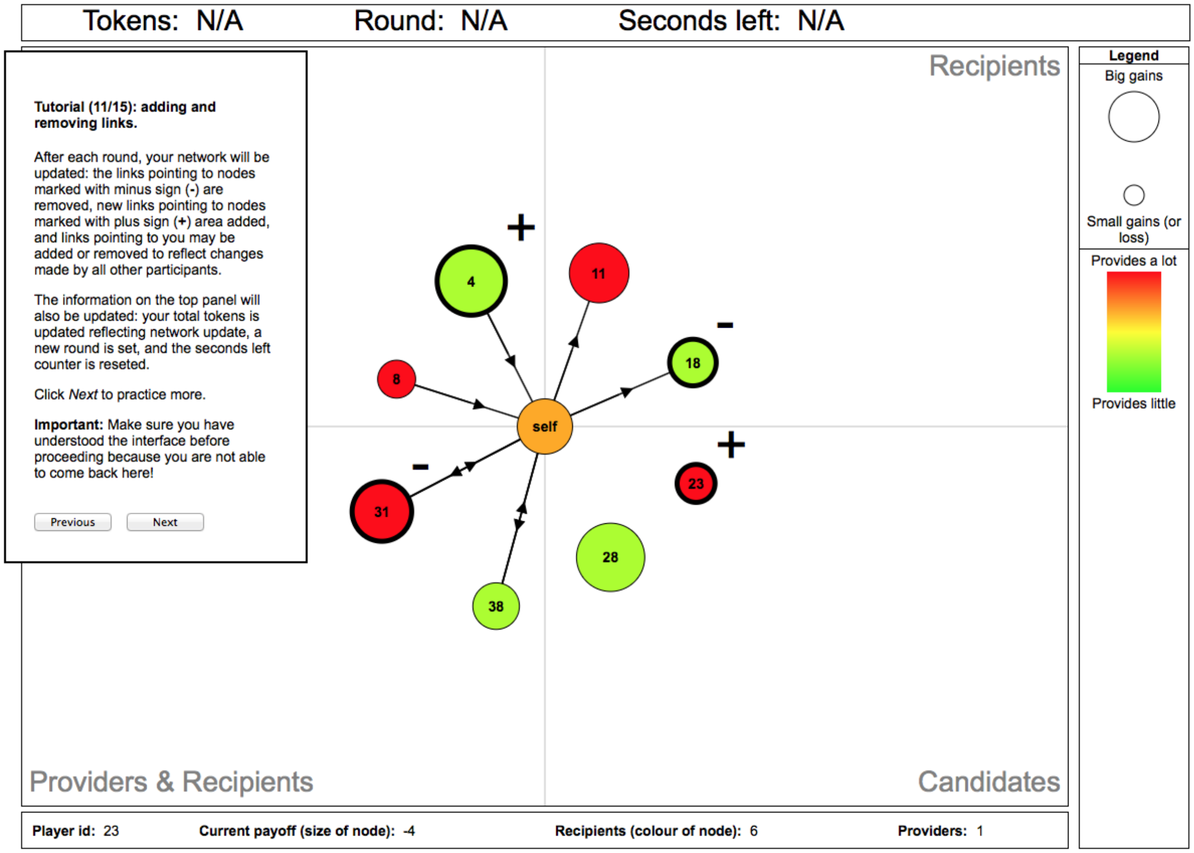

Figure R: Tutorial 11/15

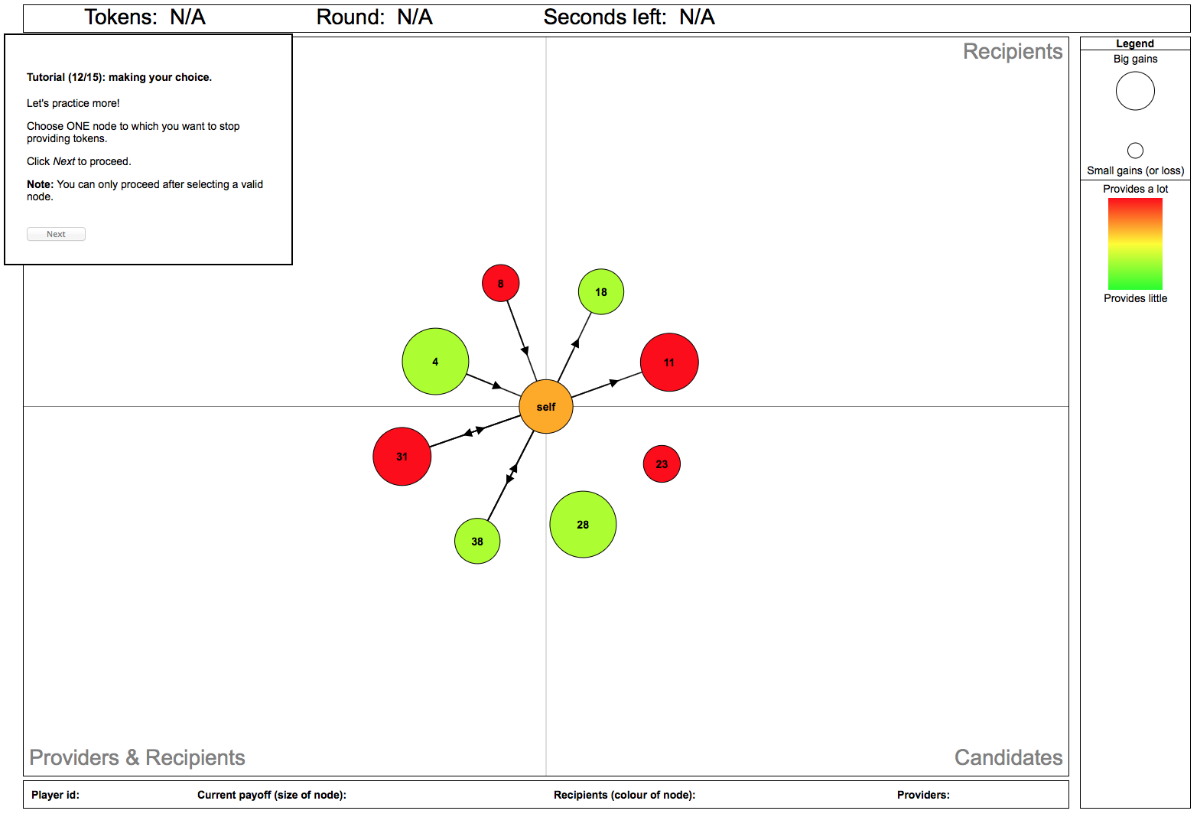

Figure S: Tutorial 12/15

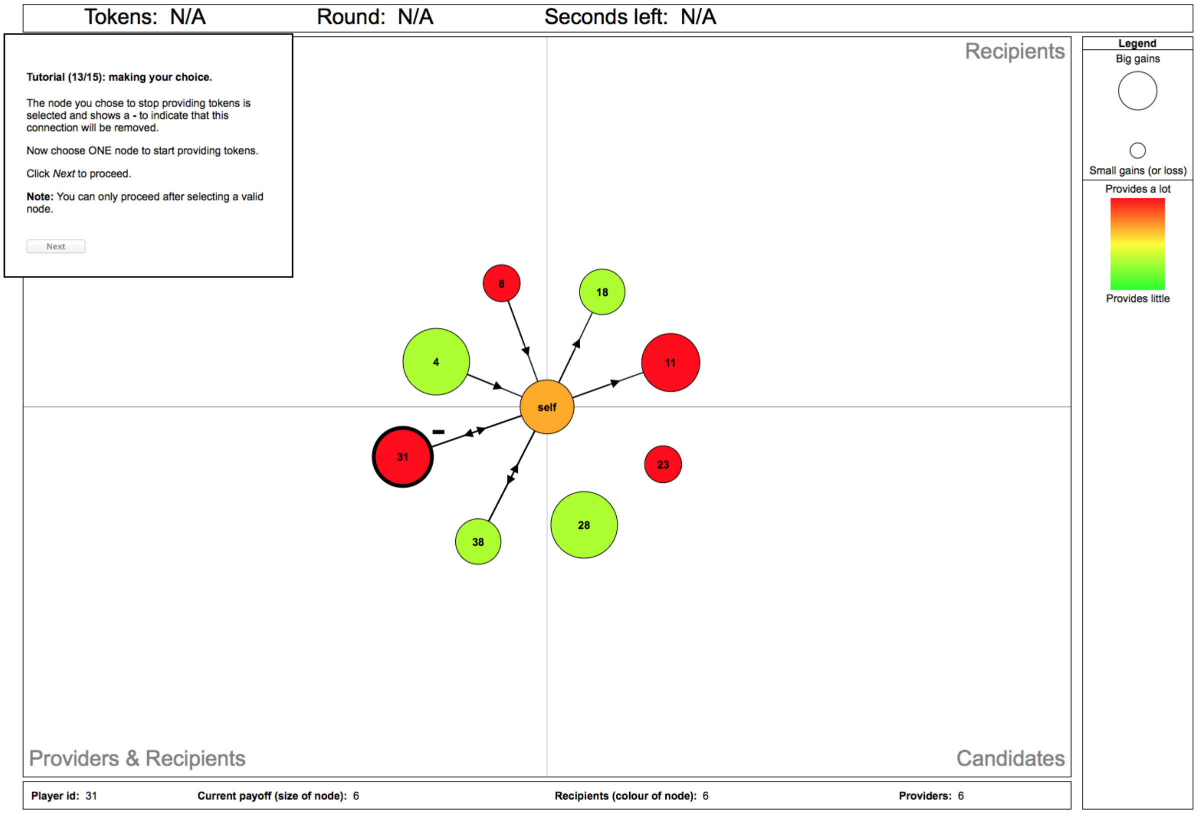

Figure T: Tutorial 13/15

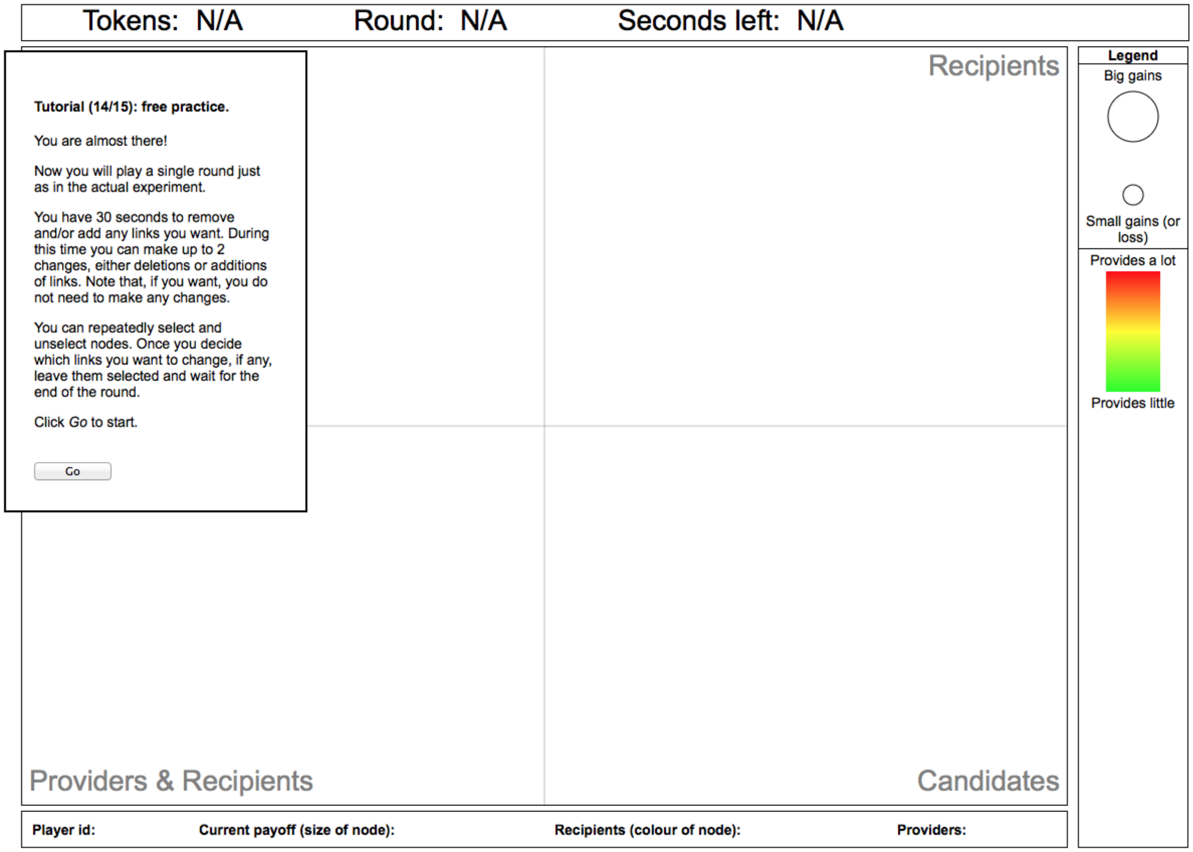

Figure U: Tutorial 14/15

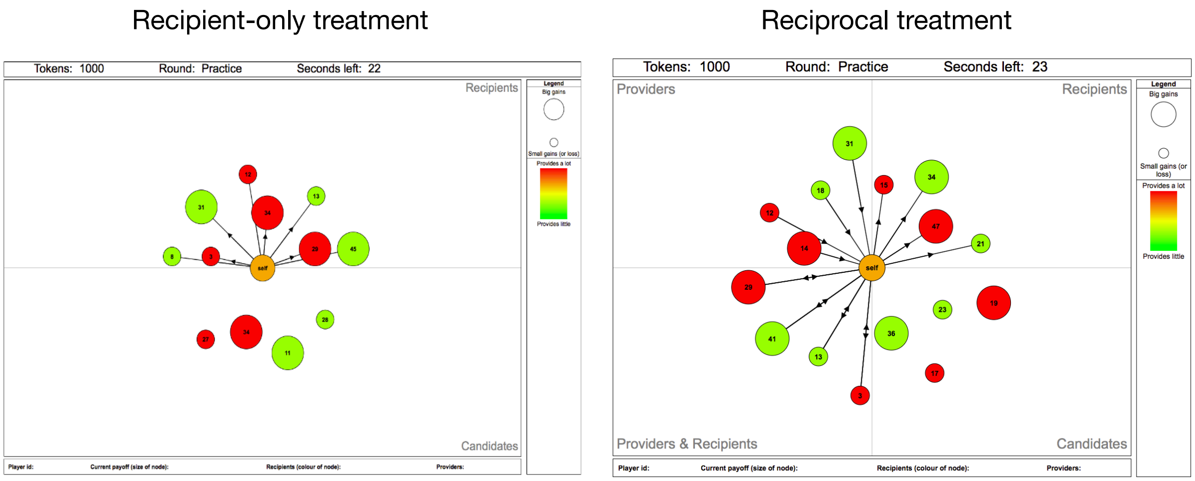

Figure V: Last practice in the tutorial for each treatment.

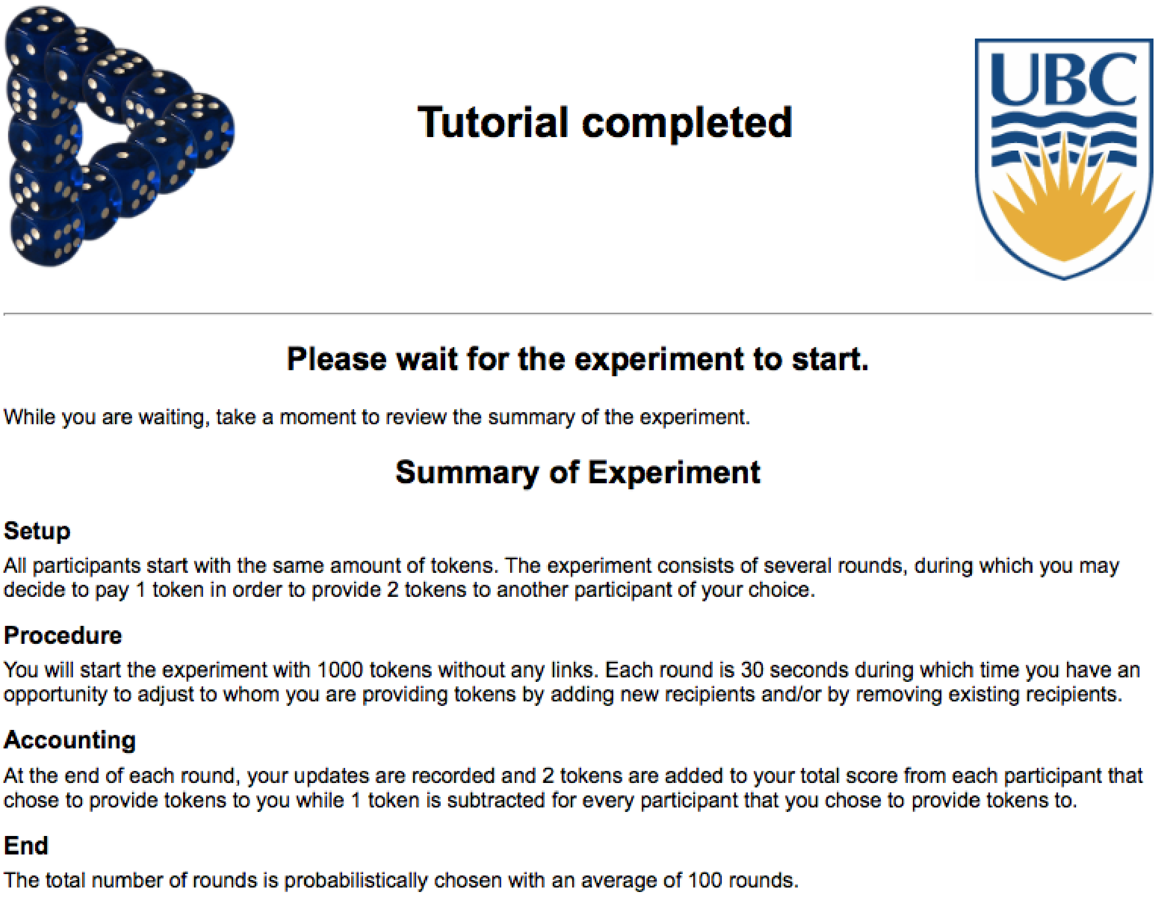

Figure W: Tutorial end

D Stationary regime

In Fig. X we show the time evolution of the normalized generosity averaged over each session separately. The average normalized generosity increases in the first rounds and reaches a regime where values are approximately stationary.

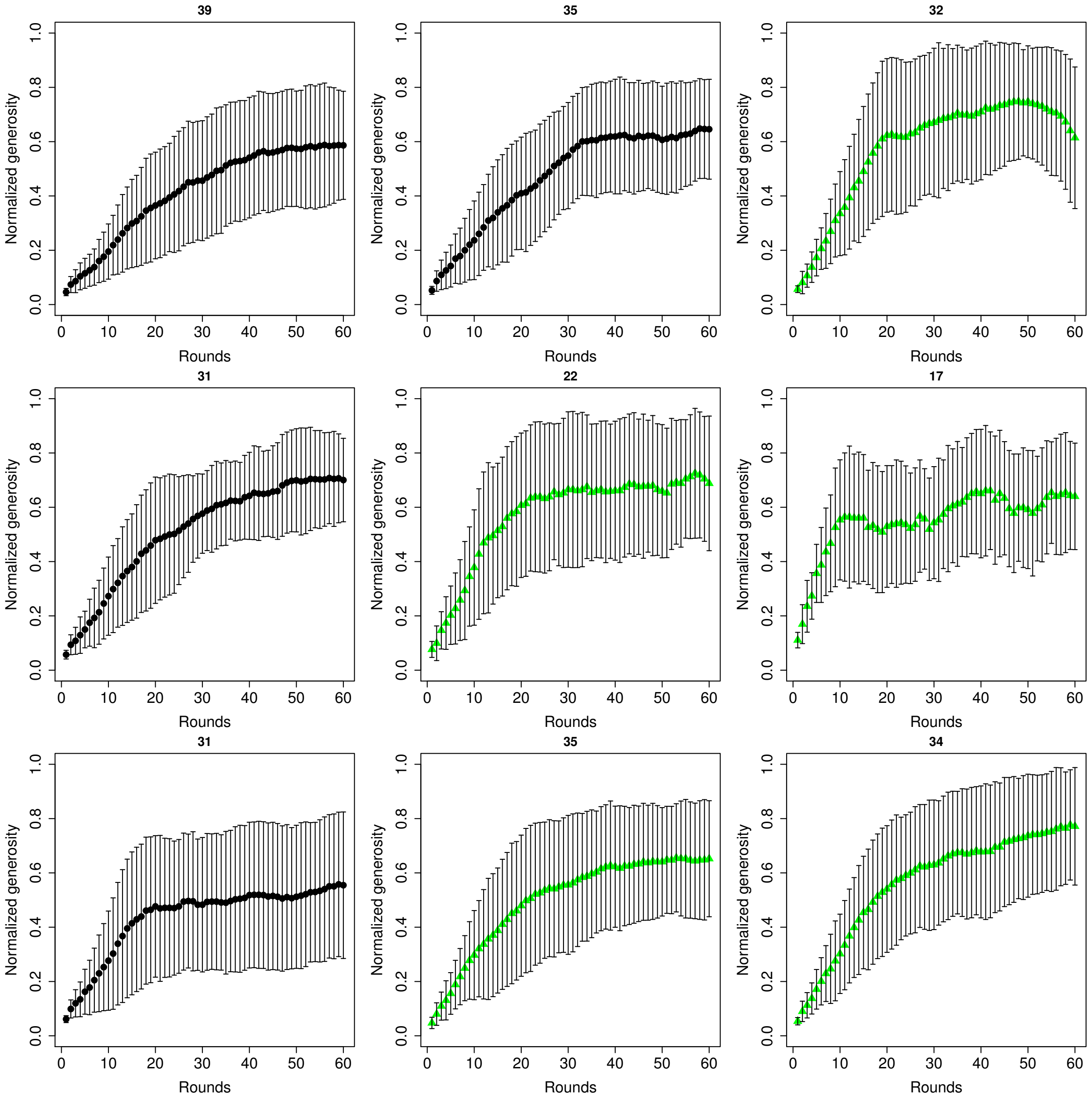

Figure X: **Normalized average generosity.** Time evolution of the normalized generosity averaged over each session separately. The number of participants is shown at the top of each. Recipient-only treatment is in black circles and the reciprocal treatment is in green triangles.

To quantify stationarity, we looked at the numerical derivative of the average generosity, defined as

$$g'(t) = g(t+1) - g(t),$$

where $g(t)$ is the average number of recipients at round t. Since each player can increase his own generosity at most by 2 links per round, the maximum derivative is 2. Note that we consider the actual number of recipients instead of the normalized version, because the maximum derivative is the same in

all session. In Fig. Y we show the derivative of the average generosity for all sessions. In principle, we say a round is in the stationary regime if the absolute value of the derivative is less than 10% of the maximum. With this definition, the stationary regime of each session starts at different rounds. To simply the analysis, we considered the stationary regime as the five last rounds of all sessions. The results do not change, as long as we are in the stationary regime of all sessions.

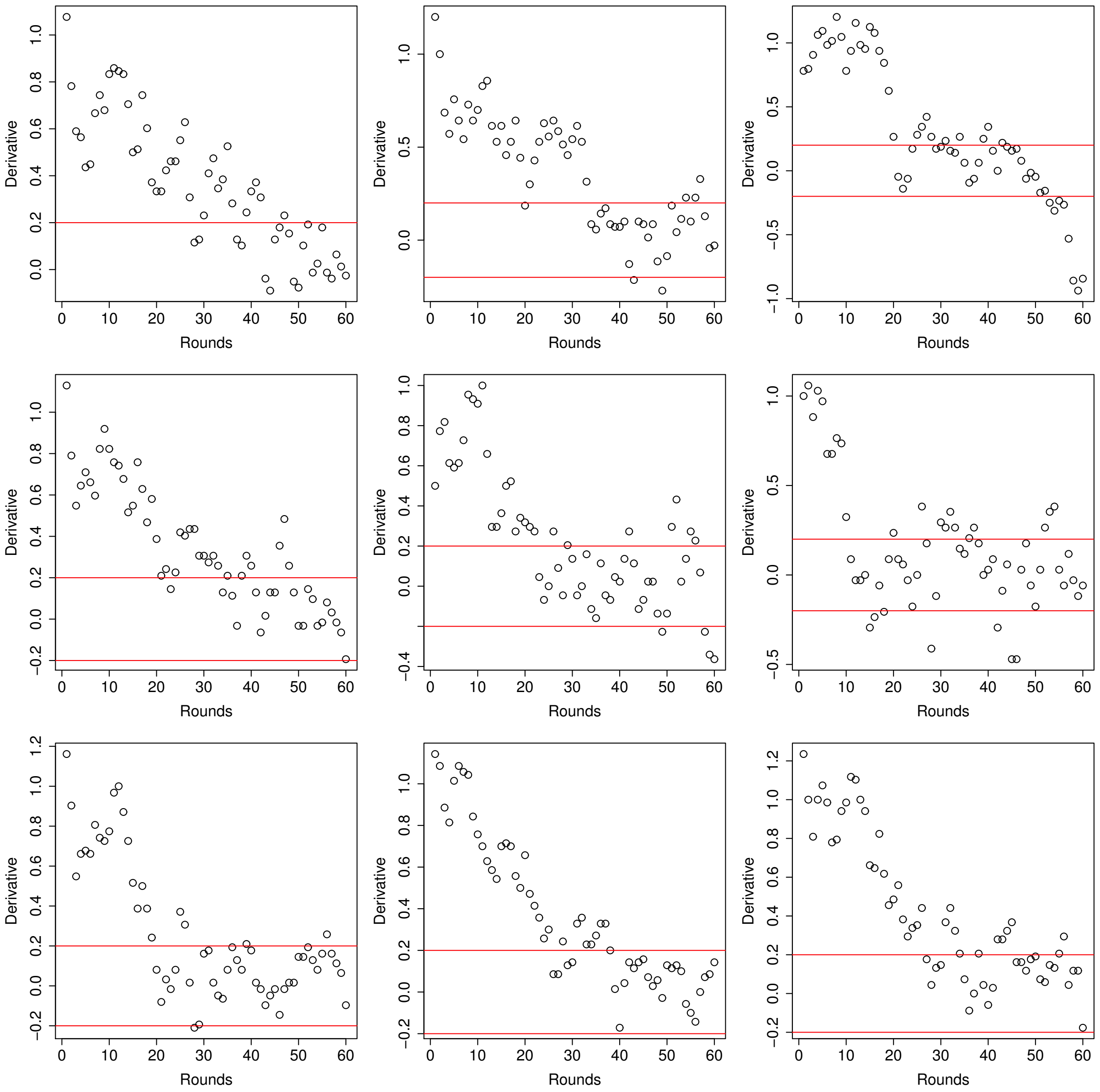

Figure Y: **Numerical derivative of the number of recipients**. Read lines delimit the area where the absolute value is less than 0.2.

E Links removed from reciprocals

In Tab S1 we show the joint distribution of removed links to reciprocals in terms of the relative generosity and relative payoff. Most links, 55%, were were removed from more successful *and* more generous targets. In this table we included the link update events where the target is as generous and as successful as the focal player.

	Less Generous	Equally Generous	More Generous
More Successful	0.095	0.024	0.550
Equally Successful	0.016	0.005	0.033
Less Successful	0.152	0.006	0.118

Table A: Joint distribution of removed links to reciprocals in term of relative payoff and relative generosity.

F Individual preference analysis

Link update events – link additions and link deletions – can be characterized in terms of relative payoff and relative generosity. Excluding the first 10 rounds, each participant can update up to 100 links during the whole experiment (up to 2 links in each one of the 50 rounds). The first 10 rounds are not taken into account because participants start as disconnected nodes and samples of providers and recipients are not available yet. Individual preferences can be defined in terms of the most frequent type of link update effectuated by an individual. For example, if most of the time a participant adds links to more generous targets, then we say that this participant prefers to add to more generous targets. In terms of generosity and success, we can define the following four preferences:

1. I prefer to add to (more | less) generous targets.
2. I prefer to remove to (more | less) generous targets.
3. I prefer to add to (more | less) successful targets.
4. I prefer to removed to (more | less) successful targets.

In the reciprocal treatment, we can also define preferences in term of target position: providers, recipients, reciprocals, and candidates. We say that a participant exhibits a preference if links are updated accordingly to this preference in more that 50% of the rounds. To assess statistical significance, we used binomial test to compare the observed frequency against a randomly expect frequency of 50%.

Figure Z shows the distribution of preferences in both treatments. The white bars shows the fraction of participants that do not exhibit any significant preference. For those exhibiting significant preferences, we observed broad diversity of preferences. In both treatments, the dominant preference for link deletion is to remove links to more successful targets and the dominant one for link addition is to add links to less successful targets, Fig. Z-b and Z-d. This dominance is preserved in the reciprocal treatment, where participants can distinguish between providers, recipients, reciprocators, and candidates. The influence of generosity on link addition do not show any dominant trait: Participants are evenly split between those that prefer to add to more generous targets and those that prefer to add to less generous targets in both treatments, Fig. Z-a and Fig. Z-c. In contrast, the influence of generosity on link deletion exhibits

interesting differences between treatments. In the recipient-only treatment, participants prefer to remove from less cooperative targets, Fig. Z-a. In the reciprocal treatment, the deletion of links depends on whether the target is reciprocating or not: if the target is a reciprocator, links are removed if targets are more generous, otherwise links are removed if targets are less generous Fig. .

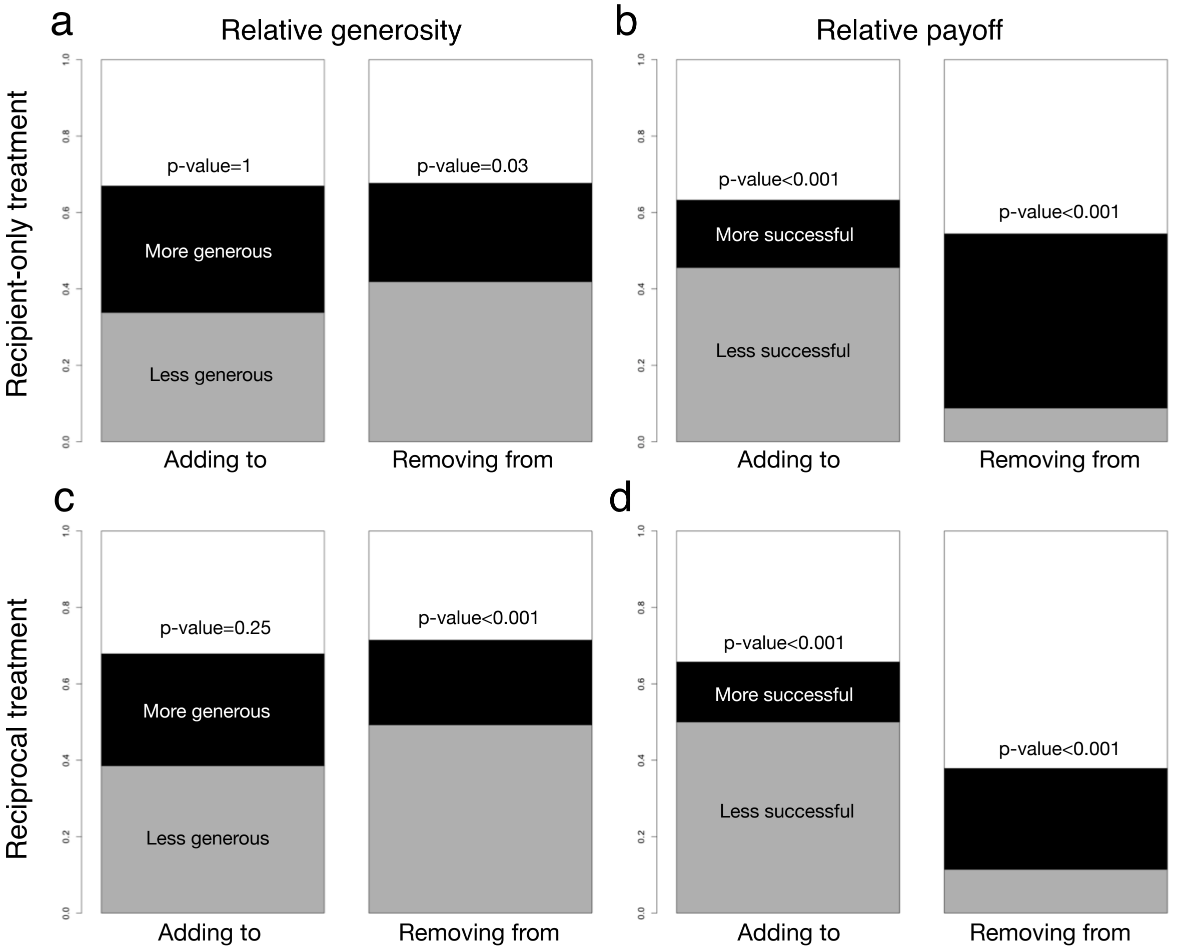

Figure Z: Individual preferences in terms of relative generosity and relative payoff. The height of the bars (black, grey, and white) represents the proportion of participants relative to the total population in each treatment. **(a)** In the recipient-only treatment, the number of participants that prefer to add to more generous is equal to the number that prefer to add to less generous models. There is a slightly larger number of individuals removing from less generous. **(b)** The number of individuals adding to less successful is larger than the number adding to more successful. The number of individuals removing from more successful is larger. **(c)** In the reciprocal treatment, the number of participants that prefer to add to more generous is roughly equal to the number that prefer to add to less generous models. **(d)** Similar to the recipient-only treatment, the number of participants adding to less successful and removing from more successful targets is the largest among those adopting consistent preferences. The p-values refer to the binomial test used to compare the proportions represented by the grey and black bars against the null hypothesis of equal proportions.

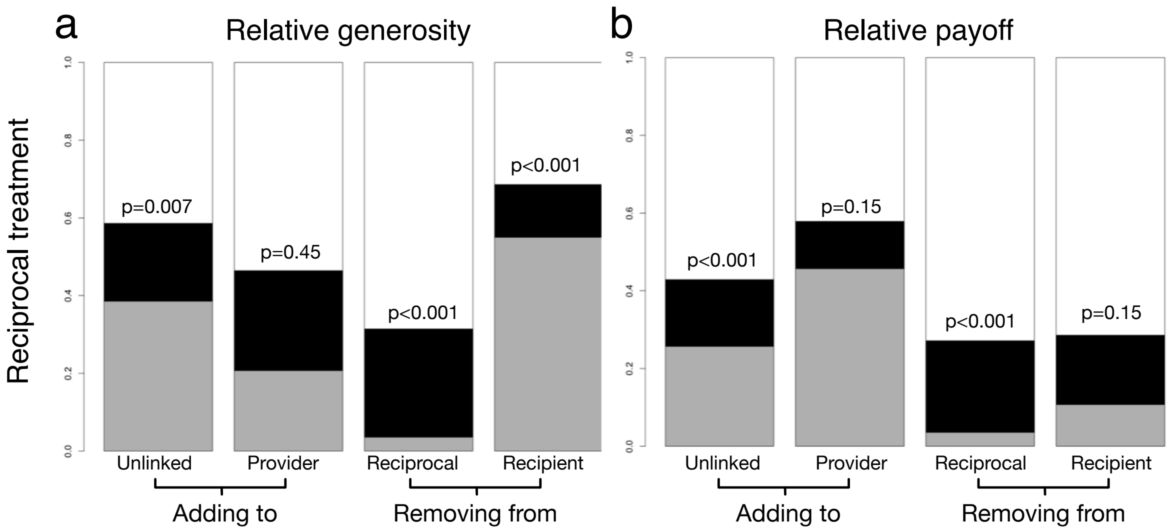

Figure : Individual preferences in terms of generosity and payoff differences for the reciprocal treatment. Preferences are defined in terms of the target position as provider, recipient, reciprocator, or candidate. The p-values refer to the comparison between the grey and black bars.

Preferences could include interacting terms, for example, 'I prefer to add to more generous and more successful targets' or 'I prefer to add to more generous and more successful targets AND to remove to to less generous and less successful targets '. However, the partition of the link event sample into these more detailed classes decreases sample size, leading to non significant statistics.
